# Targeting GALNT7 Disrupts the TAZ O‐GalNAcylation Feedback Loop to Suppress Gallbladder Cancer Progression

**DOI:** 10.1002/advs.76490

**Published:** 2026-07-11

**Authors:** Peng Qiu, Ming Zhang, Yunxiang Feng, Yibo Deng, Kai Zhao, Xiangyu Li, Yun Lu, Li Tian, Tao Yang, Wei Yao, Jianming Wang, Zhengdong Deng

**Affiliations:** ^1^ Division of Hepato‐Pancreato‐Biliary Surgery Tongji Hospital Tongji Medical College Huazhong University of Science & Technology Wuhan Hubei P. R. China; ^2^ Hubei Key Laboratory of Hepato‐Pancreato‐Biliary Diseases Wuhan Hubei P. R. China; ^3^ Clinical Medicine Research Centre for Hepatic Surgery of Hubei Province Wuhan Hubei P. R. China; ^4^ Clinical Medicine Research Centre for Pancreatic Surgery of Hubei Province Wuhan Hubei P. R. China; ^5^ Department of Thoracic Surgery Affiliated Tongji Hospital Tongji Medical College Huazhong University of Science and Technology Wuhan Hubei P. R. China; ^6^ Department of Geriatrics Affiliated Tongji Hospital Tongji Medical College Huazhong University of Science and Technology Wuhan Hubei P. R. China; ^7^ Department of Pediatric Surgery Affiliated Tongji Hospital Tongji Medical College Huazhong University of Science and Technology Wuhan Hubei P. R. China; ^8^ Department of Hepatobiliary Surgery Renmin Hospital of Wuhan University Wuhan Hubei P. R. China; ^9^ Department of Oncology Affiliated Tongji Hospital Tongji Medical College Huazhong University of Science and Technology Wuhan Hubei P. R. China; ^10^ Affiliated Maternal and Child Health Hospital of Hubei Province Tongji Medical College Huazhong University of Science and Technology Wuhan Hubei P. R. China

**Keywords:** gallbladder cancer, GALNT7, O‐GalNAcylation, olaparib, TAZ, therapeutic target

## Abstract

Gallbladder cancer (GBC) is a lethal malignancy with limited therapeutic options and dismal prognosis. Identifying the factors driving GBC progression is crucial for developing potent preventive and therapeutic approaches. Here, using quantitative proteomics, we identify GALNT7 as the most significantly upregulated glycosyltransferase in GBC tissues, associated with adverse clinical outcomes. Mechanistically, GALNT7 physically interacts with TAZ and catalyzes O‐GalNAcylation at Ser307, thereby inhibiting K48‐linked ubiquitination through the recruitment of the deubiquitinase USP7 and stabilizing the TAZ protein. This post‐translational modification promotes TAZ accumulation and subsequent activation of TEAD1‐mediated transcription, which in turn upregulates GALNT7 expression, thereby establishing a self‐reinforcing oncogenic feedback loop. Disruption of TAZ O‐GalNAcylation via the S307A mutation abrogates its oncogenic activity and attenuates GALNT7‐driven tumor progression. Importantly, structure‐based drug‐repurposing screens identified the PARP inhibitor Olaparib as a direct GALNT7 antagonist that effectively inhibits TAZ O‐GalNAcylation and demonstrates potent anti‐tumor efficacy against GBC both in vitro and in vivo. Collectively, our findings reveal a glycosylation‐dependent regulatory axis that drives GBC progression and establish GALNT7‐TAZ signaling as a tractable therapeutic target.

## Graphical abstract

Aberrant GALNT7‐mediated O‐GalNAcylation stabilizes TAZ to drive gallbladder cancer progression through a feed‐forward transcriptional loop. Structure‐based screening identifies Olaparib as a potent GALNT7 antagonist that disrupts this oncogenic axis, providing an immediate therapeutic strategy for this aggressive malignancy.

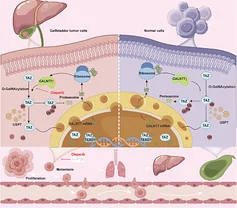

## 1 Background

Gallbladder cancer (GBC) is a highly aggressive malignancy associated with poor survival outcomes, primarily attributable to late‐stage diagnosis and rapid disease progression [1, 2]. Despite advances in surgical techniques and adjuvant therapies, clinical outcomes remain suboptimal, largely due to the longstanding under‐prioritization of GBC research. Its molecular pathogenesis remains poorly characterized compared with that of hepatocellular carcinoma, resulting in a paucity of actionable targets and clinically translatable therapeutics [3, 4]. Consequently, elucidating the molecular mechanisms underlying GBC progression is an urgent unmet need, with the potential to uncover novel therapeutic vulnerabilities and improve patient prognosis.

Post‐translational modifications (PTMs) substantially expand the functional diversity of the proteome and regulate nearly all aspects of cellular homeostasis. Among these, glycosylation is the most abundant and chemically diverse PTM, orchestrating a dynamic ‘glyco‐code’ that governs protein folding, stability, intracellular trafficking, and protein–protein interactions [5]. Dysregulation of glycosyltransferases or glycosidases is increasingly recognized as a fundamental hallmark of cancer, promoting oncogenic signaling, immune evasion, and metastatic progression [6]. Within the glycosylation landscape, mucin‐type O‐linked glycosylation (O‐GalNAcylation) is initiated by a family of twenty polypeptide N‐acetylgalactosaminyl‐transferases (GALNTs) that catalyze the transfer of N‐acetylgalactosamine (GalNAc) to serine or threonine residues [7]. Accumulating evidence implicates aberrant O‐GalNAcylation in the pathogenesis of diverse diseases, including Ischemia‐reperfusion injury [8, 9], IgA nephropathy [10, 11], congenital disorders of glycosylation [12], Parkinson's disease [13], and atherosclerosis [14]. Aberrant O‐GalNAcylation is increasingly recognized as a pervasive feature of solid tumor biology. Dysregulated O‐GalNAc glycans have been documented across multiple malignancies, including bladder [15], breast [16], lung [17], prostate [18], gastric [19], pancreatic ductal adenocarcinoma [20], hepatocellular [21] and ovarian cancers [22], where they contribute to oncogenic signaling, immune evasion, drug resistance and metastasis. Taken together, these findings establish O‐GalNAcylation as a therapeutically tractable, cancer‐specific axis. Nevertheless, the role of O‐GalNAcylation in gallbladder cancer remains largely unexplored, representing a critical knowledge gap regarding its involvement in tumor initiation, local invasion, and metastatic dissemination.

In this study, we systematically investigate the initiation and progression of gallbladder cancer and demonstrate that aberrant glycosylation patterns are prevalent in GBC, with GALNT7 functioning as a key glyco‐oncogenic driver. Specifically, we identify a GALNT7–TAZ S307 O‐GalNAcylation–USP7 feed‐forward axis that drives GBC proliferation and metastasis, and demonstrate that disruption of this ‘glyco‐code’ offers pre‐clinical proof‐of‐concept for a therapeutically tractable strategy against this invariably fatal malignancy.

## 2 Results

### 2.1 Proteomics Prioritizes GALNT7 as the Most Up‐Regulated Glycosylation Enzyme in GBC

To delineate the molecular mechanisms underlying gallbladder cancer (GBC), we conducted 4D label‐free quantitative proteomic profiling of five paired GBC and adjacent non‐tumor tissues. This analysis quantified 4,403 proteins. Among these, 299 proteins exhibited significant differential abundance: 204 were up‐regulated and 95 were down‐regulated in tumor vs. adjacent non‐tumor tissues. Furthermore, 82 proteins were unique to tumor tissues and two were exclusive to non‐tumor specimens (Table S1). Up‐regulated proteins were significantly enriched in glycosylation‐associated pathways, including Various types of N−glycan biosynthesis, N−Glycan biosynthesis and Other types of O−glycan biosynthesis (Figure 1A). Focusing on the glycosylation subset, we identified GALNT7—a glycosyltransferase—as the most prominently over‐expressed glycosylation‐related protein in GBC tissues relative to matched non‐tumor controls (Figure 1B). Subsequent validation using quantitative real‐time PCR (qRT‐PCR) and Western blotting in an expanded cohort of 16 paired samples confirmed elevated GALNT7 expression at both protein and mRNA levels (Figure 1C,D). Concordant results were obtained from two independent GEO datasets (Figure 1E). Immunohistochemistry (IHC) analysis of 100 GBC tissues and 62 cholecystolithiasis specimens further revealed significantly higher GALNT7 expression in malignant vs. benign gallbladder epithelium (Figure 1F). Overall, these data establish GALNT7 as consistently over‐expressed in GBC. To assess the clinical relevance of GALNT7, we correlated its expression with clinicopathological parameters in GBC patients. High GALNT7 expression was significantly associated with liver metastasis and shortened overall survival (Table S5 and Figure 1G). Moreover, both univariate and multivariate Cox regression analyses identified GALNT7 expression as an independent predictor of poor prognosis in GBC (Figure 1H,I and Table S6). These results collectively suggest that GALNT7 is not only pathologically associated with the aggressiveness of GBC but also serves as a clinically relevant biomarker for patient prognosis.

**FIGURE 1:**
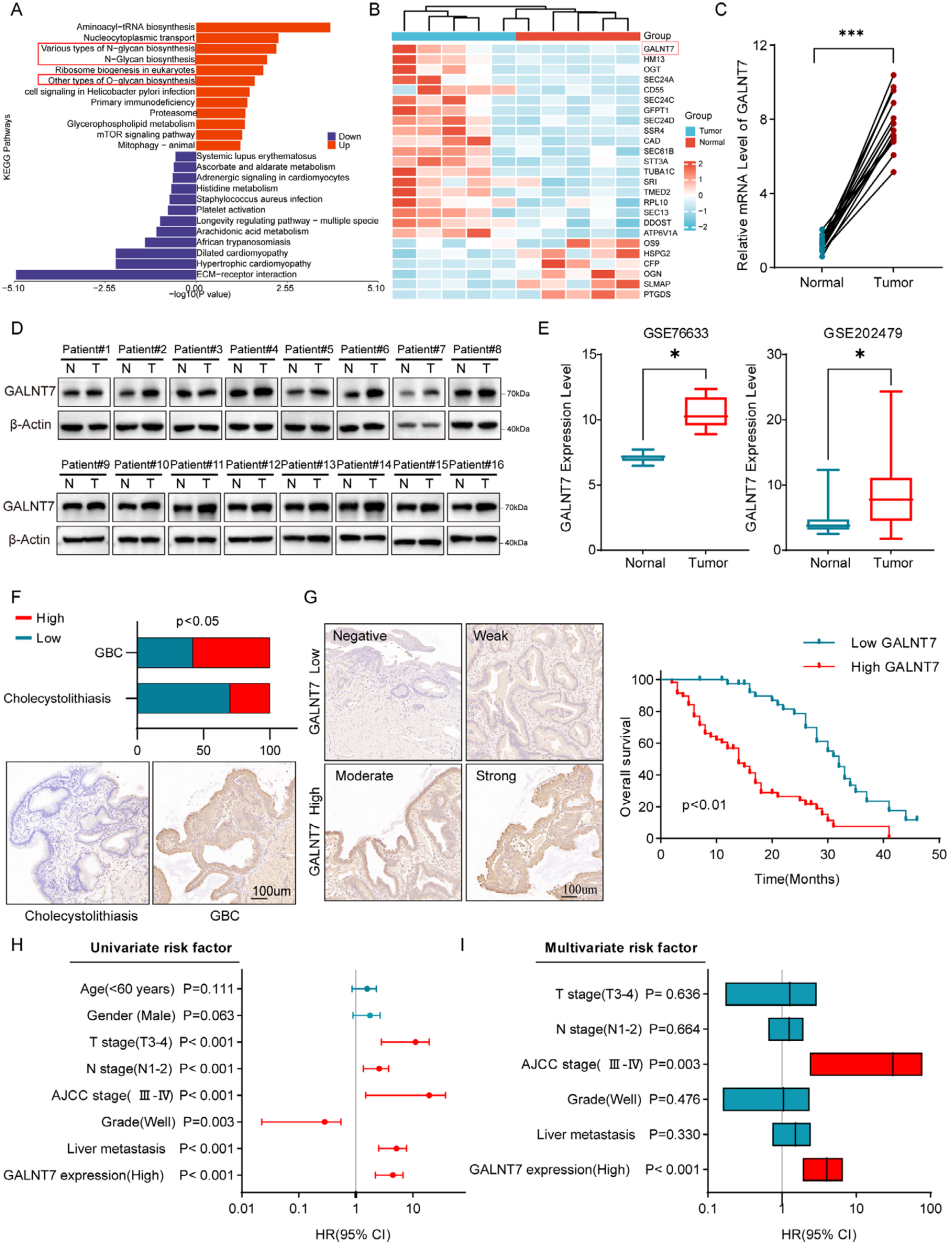
Proteomics prioritizes GALNT7 as the most up‐regulated glycosylation enzyme in GBC. (A)Enrichment‐analysis bar plot of 4D‐label‐free quantitative proteomics data from five GBC–adjacent non‐tumor tissue pairs show the significance of differentially regulated biological pathways. (B) Heatmap depicting differentially expressed glycosylation‐related proteins identified by proteomics. (C–D) Comparison of GALNT7 mRNA (C) and protein (D) expression between tumor and adjacent non‐ tumor tissues in the Tongji GBC cohort (N = 16). (E) The analysis of mRNA levels of GALNT7 in GBC and para‐carcinoma normal tissues was conducted using GEO dataset, including GSE76633 and GSE202479. (F) Images showing representative IHC staining of GALNT7 in GBC tissues (n = 100) and cholecystolithiasis (n = 62), along with quantification based on IHC scores. Scale bar, 100 µm. (G) Illustrative IHC staining images showing various scores based on intensity and percentage of stained cells and Kaplan–Meier curves show significantly longer overall survival in the low‐GALNT7 subset of the Tongji GBC cohort. Scale bar, 100 µm. (H‐I) Cox regression analyses, both univariate (H) and multivariate (I), were conducted on patients with GBC. Data are represented as means ± SD in the bar graphs. *: p < 0.05, **: p < 0.01, ***: p < 0.001. Two‐tailed paired Student's *t*‐test (C); two‐tailed unpaired Student's *t*‐test (E); chi‐square test (D); log‐rank test for survival analysis (G).

### 2.2 GALNT7 Promotes the Proliferation, Invasion, and Migration of GBC

Given the association between high GALNT7 expression and adverse clinical outcomes, we systematically examined its functional impact on GBC cell behavior using in vitro and in vivo models. Stable GALNT7 knockdown and overexpression cell lines were generated in NOZ and GBC‐SD cells using lentiviral transduction (Figure 2A). CCK‐8 assays revealed that GALNT7 depletion attenuated proliferation, whereas its overexpression significantly enhanced growth in both cell lines (Figure 2B). Colony formation assays demonstrated a direct correlation between GALNT7 expression and clonogenic capacity (Figure 2C and Figure S1A). In subcutaneous xenograft models, GALNT7 silencing significantly impaired tumor growth and reduced final tumor weight, whereas GALNT7 overexpression exerted the opposite effects (Figure 2D–F). We next evaluated the role of GALNT7 in GBC invasion and metastasis using cellular assays and an experimental lung metastasis model. Transwell assays revealed that GALNT7 depletion markedly attenuated invasion and migration, whereas its overexpression potentiated both processes (Figure 2G,H and Figure S1B). Wound healing assays corroborated these findings, showing impaired closure upon GALNT7 knockdown and accelerated migration following overexpression (Figure 2I and Figure S1C). In an experimental lung metastasis model, GALNT7 silencing significantly reduced the number of pulmonary metastatic nodules, whereas overexpression markedly increased metastatic burden (Figure 2J–L). In conclusion, these data establish GALNT7 as a central driver of GBC aggressiveness by promoting both tumor growth and metastatic dissemination.

**FIGURE 2:**
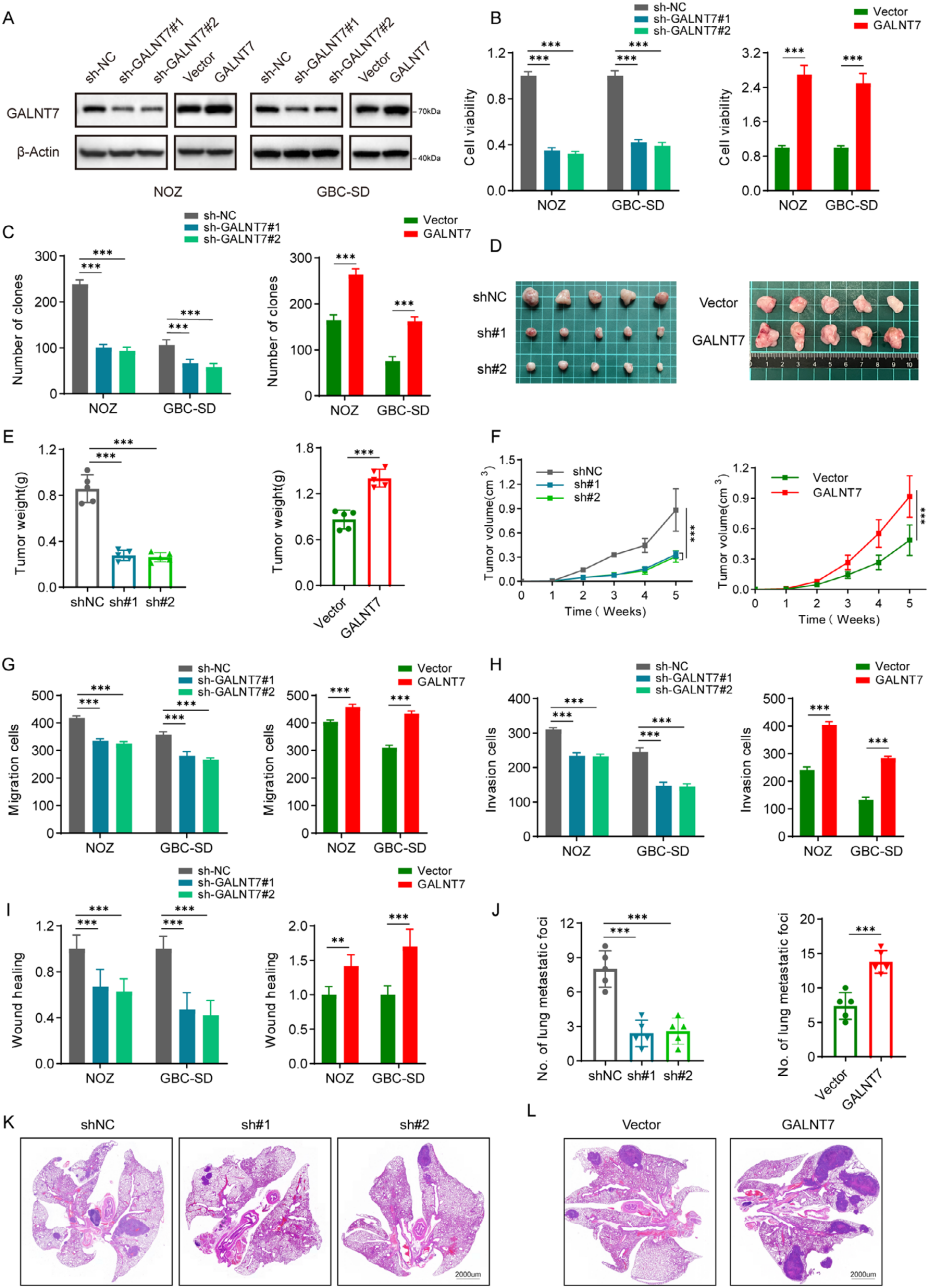
GALNT7 promotes GBC progression in vitro and in vivo. (A) Western blot analysis of GALNT7 expression post GALNT7 knockdown and overexpression in two GBC cell lines (NOZ and GBC‐SD). (B) CCK‐8 assays were used to assess the proliferation of GBC cell lines with stable GALNT7 knockdown and overexpression. (n = 5). (C) Colony formation assay quantification for indicated cells. (n = 3). (D) NOZ cells with GALNT7 knockdown or overexpression were subcutaneously implanted into nude mice; tumors were harvested and photographed (n = 5 per group). (E‐F) Weight (E)and volume(F)of tumors in panel(D). (G‐H) Quantification of cell migration and invasion assays for the specified cells. (n = 3). (I) Analysis of wound healing assays for the mentioned cells. (n = 3). (J‐L) Representative H&E staining in lung metastasis models (K‐L) and counting the number of lung metastatic nodules(J) (n = 5 per group). Data are represented as means ± SD in the bar graphs. *: p < 0.05, **: p < 0.01, ***: p < 0.001; two‐tailed unpaired Student's *t*‐test and one‐way ANOVA with Tukey's test(B, C,E,G‐J); two‐way ANOVA with Tukey's test (F).

### 2.3 GALNT7 Physically Interacts With TAZ and Blocks Its Proteasomal Degradation in a Glycosyltransferase Activity‐Dependent Manner

To elucidate the mechanistic basis by which GALNT7 drives GBC progression, we conducted immunoprecipitation coupled with mass spectrometry (IP‐MS) to identify GALNT7‐interacting proteins. The Hippo transcriptional co‐activator TAZ (encoded by WWTR1) emerged as one of the most enriched, high‐confidence interactors (Figure 3A, Figure S2A and Table S2). Given its well‐established oncogenic role, TAZ was selected as the primary interactor for mechanistic follow‐up. Reciprocal co‐immunoprecipitation (co‐IP) assays confirmed the GALNT7–TAZ interaction in both GBC and HEK293T cell lines (Figure 3B,C and Figure S2B). GST pull‐down assays further validated the direct and specific interaction between GALNT7 and TAZ, as both proteins bound only to their respective GST‐fused counterparts and not to GST alone (Figure 3D). High‐resolution confocal microscopy using GM130 as a Golgi marker revealed that GALNT7 exhibited strong colocalization with GM130, confirming its predominant Golgi residence. Significantly, TAZ also displayed marked colocalization with GM130 and with GALNT7, indicating that the interaction between GALNT7 and TAZ occurs primarily within the Golgi apparatus (Figure 3E and Figure S2C) and in human GBC tissue sections (Figure S2D). Domain‐mapping experiments delineated the interaction interface to TAZ residues 194–400 (T3 domain) and the ricin‐B‐type lectin domain of GALNT7 (residues 532–657) (Figure S2E). To determine the functional consequences of this interaction, we examined whether GALNT7 modulates TAZ expression. Stable GALNT7 knockdown reduced endogenous TAZ protein abundance without affecting TAZ mRNA, whereas GALNT7 overexpression markedly increased TAZ protein levels, again with no substantive change in transcript abundance (Figure 3F,G). Collectively, these data demonstrate that GALNT7 post‐transcriptionally stabilizes TAZ via direct protein–protein interaction, thereby promoting GBC progression. We next investigated whether GALNT7 increases TAZ abundance by extending its protein half‐life. Cycloheximide‐chase assays revealed that GALNT7 depletion accelerated TAZ turnover, whereas GALNT7 overexpression markedly extended its half‐life (Figure 3J–O and Figure S2G,H). In eukaryotic cells, protein turnover is primarily governed by the ubiquitin–proteasome system or the autophagy–lysosomal pathway [23]. Pharmacological interrogation showed that only the proteasome inhibitor MG132 restored TAZ levels following GALNT7 knockdown, whereas autophagy (3‐MA) or lysosomal (CQ) inhibitors were ineffective (Figure 3H,I and Figure S2F). Furthermore, ubiquitination assays showed that GALNT7 silencing increased, and its ectopic expression decreased, polyubiquitinated TAZ (Figure 3P,Q). Previous studies have shown that TAZ undergoes K48‐ and K63‐linked polyubiquitination, either of which can target it for proteasomal destruction [24, 25]. Co‐transfection experiments using wild‐type, K48‐only, or K63‐only ubiquitin mutants revealed that GALNT7 selectively removes K48‐linked ubiquitin chains from TAZ, while K63 linkages remain unaffected. This effect was abrogated when a K48R ubiquitin mutant was employed (Figure 3R). Together, these data establish that GALNT7 antagonizes K48‐linked ubiquitination of TAZ, thereby preventing its proteasomal degradation.

**FIGURE 3:**
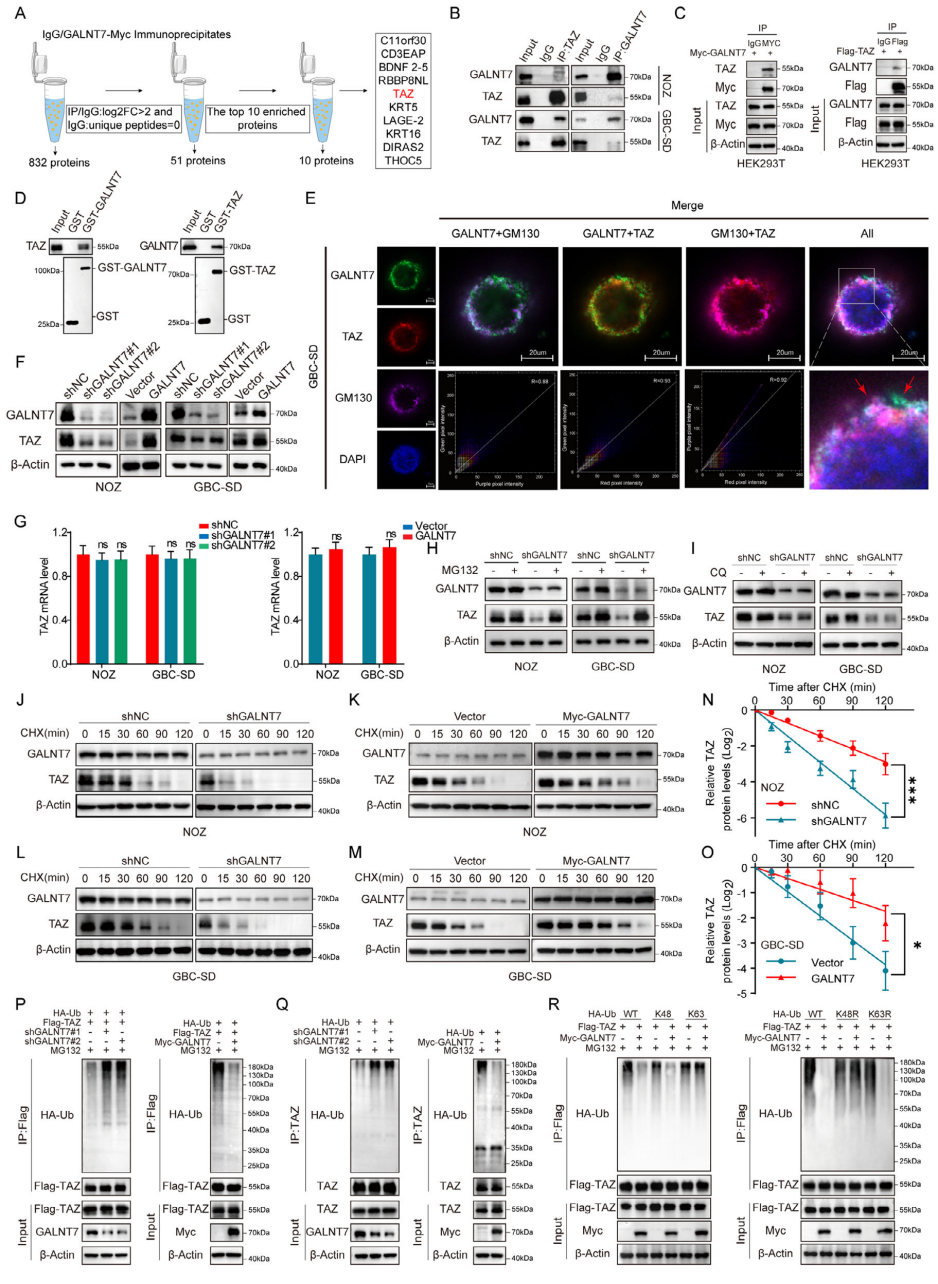
GALNT7 Interacts with TAZ and stabilizes TAZ Protein by reducing K48 ‐linked ubiquitination. (A) The flow chart of screening the protein partner of GALNT7. (B) Reciprocal Co‐IP of endogenous GALNT7 and TAZ in NOZ and GBC‐SD cells confirmed the interaction. (C) The interaction between endogenous TAZ and exogenous GALNT7 is shown in the left panel, while the interaction between endogenous GALNT7 and exogenous TAZ is depicted in the right panel. To investigate these interactions, exogenous GALNT7 or TAZ was transfected into 293T cells. Subsequently, cell lysates were prepared and subjected to co‐immunoprecipitation (Co‐IP) using specific antibodies, followed by analysis via immunoblotting. (D) GST pull‐down confirmed direct GALNT7–TAZ interaction. (E) High‐resolution confocal microscopy reveals Golgi‐localized GALNT7 partially colocalizes with TAZ. Representative confocal images of endogenous GALNT7 (green), TAZ (red), GM130 (magenta, cis‐Golgi marker), and DAPI (blue, nucleus) in GBC‐SD cells. Scale bar, 20 µm. (F‐G) Immunoblotting analysis(F) and RT‐qPCR (G) were performed to assess the protein expression of TAZ in NOZ and GBC‐SD cells with engineered to knock down or overexpress GALNT7. (H‐I) NOZ and GBC‐SD cells stably silenced for GALNT7 were exposed to MG132 (10 µM) or CQ (50 µM) for 6 h; immunoblot showed MG132 (H), but not CQ (I), restored TAZ levels. (J‐O) CHX chase (50 µM) revealed accelerated TAZ turnover upon GALNT7 knockdown and prolonged half‐life upon Myc‐GALNT7 overexpression; quantification normalized to t = 0 and β‐actin. (P‐Q) Ubiquitination assays in HEK293T cells co‐expressing HA‐Ub and shGALNT7 or Myc‐GALNT7 (MG132, 10 µM, 6 h) showed GALNT7 knockdown enhanced, whereas GALNT7 overexpression reduced, TAZ poly‐ubiquitination. (R) Ubiquitination assays with K48‐only, K63‐only, K48R or K63R ubiquitin mutants demonstrated that Myc‐GALNT7 selectively suppresses K48‐linked ubiquitination of FLAG‐TAZ. Data are represented as means ± SD in the bar graphs. *: p < 0.05, **: p < 0.01, ***: p < 0.001; two‐way ANOVA with Tukey's test (N‐O).

To determine whether GALNT7‐mediated TAZ stabilization requires its glycosyltransferase catalytic activity, we generated a catalytically inactive mutant (GALNT7‐Mut). VVA lectin blotting of whole‐cell lysates revealed that GALNT7 knockdown substantially reduced global O‐GalNAcylation levels across the GBC proteome compared to shNC controls, confirming GALNT7 as a major contributor to the cellular O‐GalNAc landscape (Figure S3A). Conversely, overexpression of GALNT7‐WT markedly elevated global O‐GalNAc levels, whereas GALNT7‐Mut showed no significant effect on the cellular O‐GalNAc glycoproteome (Figure S3B). Besides, GALNT7‐WT significantly increased TAZ protein levels in both NOZ and GBC‐SD cells, whereas GALNT7‐Mut failed to do so, indicating that catalytic activity is essential for TAZ stabilization. We next examined whether GALNT7‐Mut affects TAZ protein stability. CHX chase assays demonstrated that GALNT7‐WT significantly prolonged TAZ half‐life, but GALNT7‐Mut did not (Figure S3D–G). Moreover, ubiquitination assays showed that GALNT7‐WT reduced TAZ ubiquitination compared to GALNT7‐Mut (Figure S3H), indicating that catalytic activity is required for preventing TAZ degradation. Functionally, GALNT7‐Mut lost the ability to promote GBC cell proliferation, colony formation, migration, invasion and wound healing (Figure S3I–P). Collectively, these findings establish that GALNT7 stabilizes TAZ and drives GBC progression strictly through its glycosyltransferase catalytic activity.

### 2.4 GALNT7 Induces O‐GalNAcylation of Serine 307 on TAZ

Having established that GALNT7 stabilizes TAZ through its glycosyltransferase activity, we next delineated the specific modification mechanism. Given that GALNT7 is an O‐GalNAc transferase, we hypothesized that it directly modifies TAZ. Ectopic Flag‐TAZ in HEK293T cells exhibited increased O‐GalNAcylation upon GALNT7 overexpression (Figure 4A). Reciprocally, GALNT7 knockdown decreased endogenous TAZ O‐GalNAcylation in GBC cells. To further establish that this modification depends on enzymatic activity, we compared GALNT7‐WT and the catalytically inactive mutant GALNT7‐Mut. GALNT7‐WT markedly enhanced TAZ O‐GalNAcylation, whereas GALNT7‐Mut failed to do so, confirming that TAZ is a bona fide O‐GalNAc substrate of GALNT7 and that the glycosylation strictly requires catalytic activity (Figure 4B). To address whether TAZ O‐GalNAcylation undergoes further elongation with additional sugars, we examined the glycosylation status using lectins specific for elongated structures. We performed immunoprecipitation of TAZ followed by lectin blotting with Sambucus nigra agglutinin (SNA), which recognizes α2‐6 linked sialic acid, and Peanut agglutinin (PNA), which binds to the T antigen (Galβ1‐3GalNAc) formed by β1‐3 galactosyltransferase‐mediated elongation of the Tn antigen. Notably, neither SNA nor PNA showed detectable binding to TAZ immunoprecipitates under conditions where VVA lectin readily detected TAZ O‐GalNAcylation (Figure 4C and Figure S4A). These results demonstrate that TAZ O‐GalNAcylation is restricted to the initial Tn antigen structure (GalNAcα1‐Ser/Thr) without further elongation to sialyl‐Tn or T antigen structures, indicating that GALNT7‐mediated modification of TAZ does not proceed through the canonical O‐glycan elongation pathway. IP‐MS was performed to identify the potential GALNT7‐dependent TAZ O‐GalNAcylation site using anti‐Flag immunoprecipitated samples with or without GALNT7 overexpression. The results identified that Serine 307 is the potential residue (Figure 4D and Figure S4B,C). Introduction of the S307A mutant, which cannot be O‐GalNAcylated, abrogated GALNT7‐mediated TAZ glycosylation, confirming Ser307 as the obligatory site (Figure 4E). To exclude the possibility that TAZ undergoes O‐GlcNAcylation (catalyzed by OGT, which was also identified as upregulated in GBC proteomics), we immunoprecipitated Flag‐tagged TAZ from HEK293T cells and probed with O‐GlcNAc‐specific antibody. No O‐GlcNAc signal was detected on either wild‐type TAZ or the S307A mutant, despite robust expression of both proteins (Figure S4D). These results demonstrate that TAZ is not modified by O‐GlcNAcylation, confirming that the Ser307 modification represents O‐GalNAcylation rather than O‐GlcNAcylation. A custom antibody directed against the O‐GalNAcylated S307 peptide validated specificity by dot‐blot analysis (Figure 4F). Using this antibody, we verified that GALNT7 knockdown markedly reduces TAZ S307 O‐GalNAcylation (Figure 4G). To further validate the specificity of the custom‐generated TAZ Ser307 O‐GalNAc antibody and confirm the glycan structure, we performed complementary assays. O‐glycosidase treatment of NOZ and GBC‐SD cell lysates did not eliminate the antibody recognition band (Figure S4E), consistent with the enzyme's inability to hydrolyze the unsubstituted Tn antigen (GalNAcα1‐O‐Ser/Thr). This result, combined with the absence of SNA (sialic acid) and PNA (T antigen/Core 1) binding (Figure 4C), confirms that TAZ S307 carries the simplest Tn antigen structure without further elongation. The strict specificity of the antibody for the glycosylated epitope was further confirmed by the complete absence of signal in the S307A mutant (Figure S4F). Since GALNT7 stabilizes TAZ by antagonizing K48‐linked ubiquitination, we next asked whether this stabilization requires O‐GalNAcylation at Ser307. We first used CRISPR‐Cas9 to eliminate endogenous TAZ in NOZ and GBC‐SD cells, followed by lentiviral reconstitution with empty vector, TAZ‐WT, or the O‐GalNAc‐deficient S307A mutant (Figure S4H,I). The S307A mutant displayed a significantly shorter half‐life than wild‐type TAZ in TAZ‐knockout GBC cells (Figure 4I–L). MG132 treatment completely rescued S307A‐TAZ degradation, confirming that its reduced stability reflects enhanced proteasomal turnover (Figure 4H). Additionally, we co‐transfected Flag‐ TAZ with Myc‐Ub (wild‐type, K48‐only, or K48R) into HEK293T cells. Our results showed that GALNT7 overexpression reduced the ubiquitination of WT TAZ but not the S307A mutant, while this effect was observed under both wild‐type and K48‐only ubiquitination conditions, but not under K48R conditions (Figure 4M). Cumulatively, these data establish that O‐GalNAcylation at Ser307 is required for GALNT7‐mediated stabilization of TAZ via suppression of K48‐linked ubiquitination and proteasomal degradation. Having established that Ser307 is the critical site for GALNT7‐mediated O‐GalNAcylation, we investigated whether this modification affects canonical Hippo pathway regulation. Surprisingly, GALNT7 overexpression did not significantly alter p‐TAZ Ser89 levels in either TAZ‐WT or TAZ‐S307A cells (Figure S4G), indicating that O‐GalNAcylation stabilizes TAZ independently of LATS1/2‐mediated phosphorylation. This unexpected finding suggests that glycosylation and phosphorylation regulate TAZ through spatially distinct mechanisms, with Ser307 O‐GalNAcylation promoting protein stability without interfering with the phosphorylation‐dependent cytoplasmic retention machinery.

**FIGURE 4:**
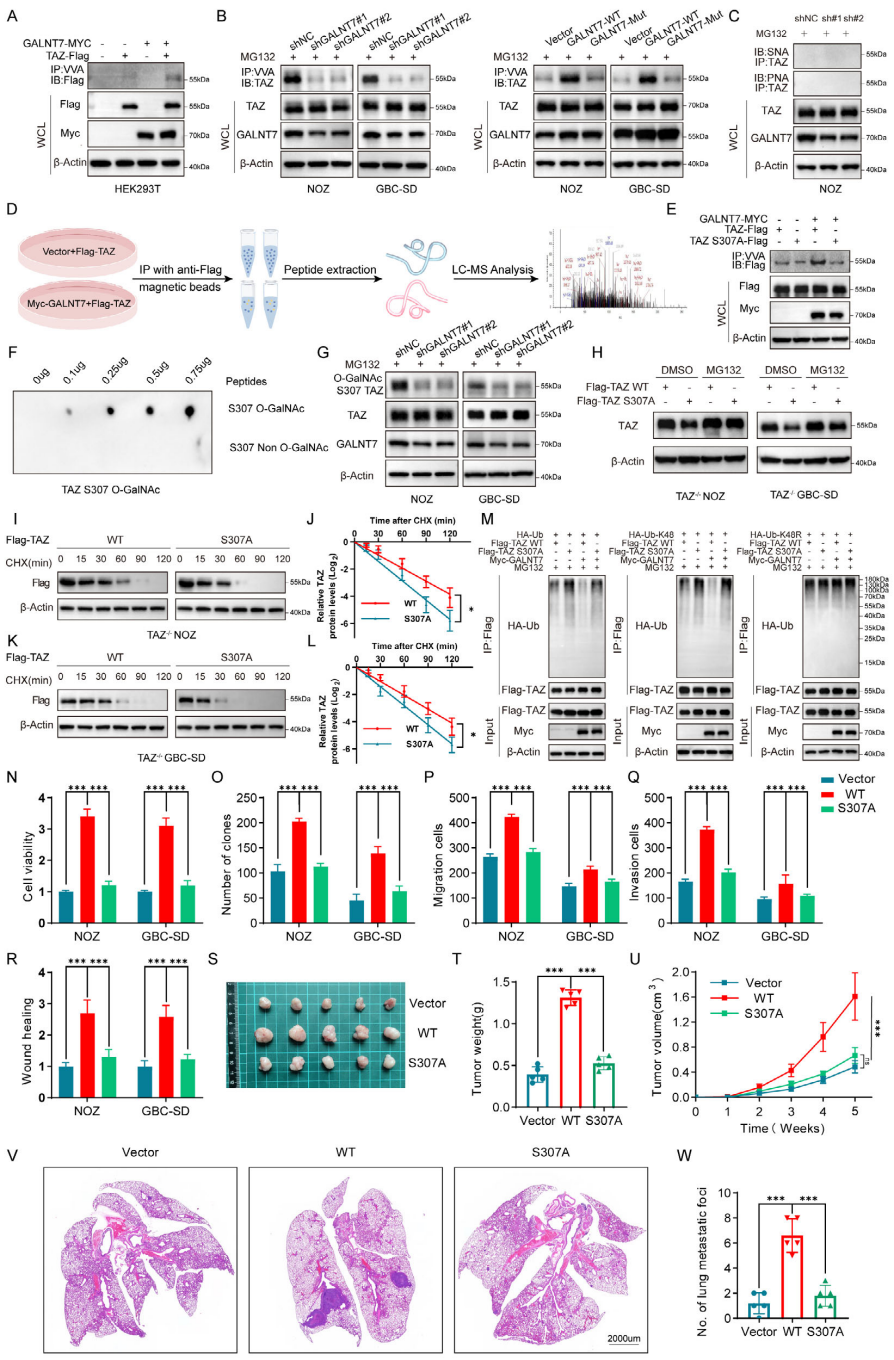
Serine residue 307 of TAZ serves as a target site for GALNT7‐mediated O‐GalNAcylation. (A‐B) O‐GalNAcylation of TAZ was assessed by Flag‐ or TAZ‐IP followed by VVA lectin blot (Tn antigen) in HEK293T and GBC cells. (C) TAZ O‐GalNAcylation does not undergo further elongation in NOZ cell line. TAZ was immunoprecipitated from NOZ cells treated with MG132 and analyzed by lectin blotting. SNA (sialic acid‐specific) and PNA (T antigen‐specific) showed no binding to TAZ, indicating the absence of glycan elongation. (D) IP‐MS workflow for mapping TAZ O‐GalNAc sites. (E) VVA pull‐down assay for the modified sites of TAZ for GALNT7. (F) Serial dilutions of the indicated peptides were spotted onto PVDF and probed with the anti–TAZ S307 O‐ GalNAcylation antibody in a dot‐blot assay. (G) WCL collected from NOZ and GBC‐SD cells with stably knockdown GALNT7 were immunoblotted with TAZ S307 specific O‐GalNAcylation antibody. (H) MG132 rescued TAZ‐S307A from degradation in NOZ and GBC‐SD Cas9/sgTAZ‐knockout cells after 6 h treatment with 10 µM MG132. (I‐L) CHX‐chase (50 µM) in NOZ and GBC‐SD TAZ‐KO cells re‐expressing WT or S307A TAZ showed accelerated degradation of the unglycosylated mutant. (M) Co‐transfection of Flag‐TAZ (WT or S307A), Myc‐GALNT7 and HA‐Ub (WT, K48‐only or K48R) followed by MG132 (10 µM, 6 h) revealed GALNT7‐dependent suppression of K48‐linked ubiquitination only on the S307 site. (N‐O) CCK‐8 assays (n = 5) and colony formation assay (n = 3) to examine the proliferation of two TAZ KO gallbladder cancer cell lines stably expressing Vector, TAZ WT, or TAZ S307A.(P‐Q) Migration and invasion capacities of the indicated cells were quantified by Transwell assays. (n = 3). (R) Quantification of wound healing assays for the indicated cells. (n = 3). (S) NOZ TAZ‐KO cells stably carrying empty vector, TAZ‐WT, or TAZ‐S307A were subcutaneously inoculated into BALB/c‐nude mice (n = 5 per group) to establish xenograft tumors. (T‐U) Tumor weight (T) and volume (U) were quantified for each group. (V)Representative H&E staining of lung metastasis models. (n = 5 per group) (W) Lung metastasis models (n = 5 per group) displayed fewer metastatic nodules in S307A and vector groups vs. WT‐TAZ. Data are represented as means ± SD in the bar graphs. *: p < 0.05, **: p < 0.01, ***: p < 0.001; two‐way ANOVA with Tukey's test (J, L, U); one‐way ANOVA with Tukey's test (N‐R, T, W).

### 2.5 TAZ S307 O‐GalNAcylation Promotes the Proliferation, Invasion, and Migration of GBC

To determine the functional consequences of TAZ Ser307 O‐GalNAcylation in GBC aggressiveness, we conducted complementary in vitro and in vivo assays. Re‐expression of wild‐type TAZ in TAZ‐knockout NOZ and GBC‐SD cells markedly enhanced proliferation relative to vector controls, as assessed by CCK‐8 and colony formation assays (Figure 4N–O and Figure S4J). In contrast, the S307A mutant showed markedly attenuated proliferation, indicating that Ser307 O‐GalNAcylation is indispensable for TAZ‐mediated growth promotion. Transwell and wound healing assays further showed that wild‐type TAZ restoration markedly restored invasive and migratory capacities, whereas the S307A mutant remained functionally inert (Figure 4P–R and Figure S4K,L). Mice engrafted with S307A‐TAZ cells developed smaller primary tumors and fewer pulmonary metastatic nodules than mice receiving WT‐TAZ cells (Figure 4S–W). These results collectively demonstrate that Ser307 O‐GalNAcylation is indispensable for TAZ‐driven proliferation, invasion, and metastasis in GBC.

### 2.6 GALNT7 Stabilizes Taz by Promoting Usp7‐Mediated Taz Deubiquitylation

To dissect how O‐GalNAcylation stabilizes TAZ, we focused on the tight regulation of TAZ protein levels by ubiquitination. We speculated that TAZ stabilization might result from the recruitment of a deubiquitinating enzyme (DUB) or the dissociation of an E3 ligase, thereby stabilizing TAZ. Using IP‐MS on anti‐Flag immunoprecipitated samples from cells overexpressing TAZ WT or TAZ S307A, we identified differentially associated proteins (Table S3). Among the known E3 ligases and DUBs interacting with TAZ, we found that USP7, a DUB, uniquely associated with TAZ WT, while MIB2, an E3 ligase, uniquely associated with TAZ S307A (Figure 5A). Co‐IP assays confirmed that USP7 bound more strongly to TAZ WT than to TAZ S307A, whereas MIB2 did not show a significant difference (Figure 5B). Further validation in two GBC cell lines revealed that overexpressing GALNT7 increased the interaction between TAZ and USP7 (Figure 5C). To confirm that the recruitment of USP7 to TAZ is dependent on the O‐GalNAcylation status at Ser307, we performed co‐immunoprecipitation assays in HEK293T cells. In the presence of GALNT7, wild‐type TAZ exhibited robust binding to USP7, whereas the glycosylation‐deficient S307A mutant showed markedly impaired interaction with USP7 (Figure S5A). This demonstrates that O‐GalNAc modification at Ser307 is required for efficient recruitment of USP7 to TAZ. To investigate how USP7 recruitment affects TAZ ubiquitination, we conducted cellular ubiquitination assays. Utilizing an independent shRNA to deplete USP7 resulted in a pronounced increase in poly‐ubiquitination for TAZ WT. This effect was notably absent in the case of the TAZ S307A mutant, highlighting the critical role of S307 in USP7‐mediated regulation of TAZ ubiquitination (Figure 5D). Similarly, the USP7 inhibitor P5091 induced comparable ubiquitination effects (Figure 5D). Notably, these effects were abolished when using a K48R ubiquitin plasmid but not a K63R plasmid, indicating the involvement of K48‐linked ubiquitination (Figure 5E). Consistent with these findings, USP7 depletion significantly affected the protein half‐life of TAZ WT but not S307A in the GBC cell lines (Figure 5F–I). The findings indicate that O‐GalNAcylation of TAZ enhances its binding to USP7, promoting deubiquitylation and stabilization of TAZ.

**FIGURE 5:**
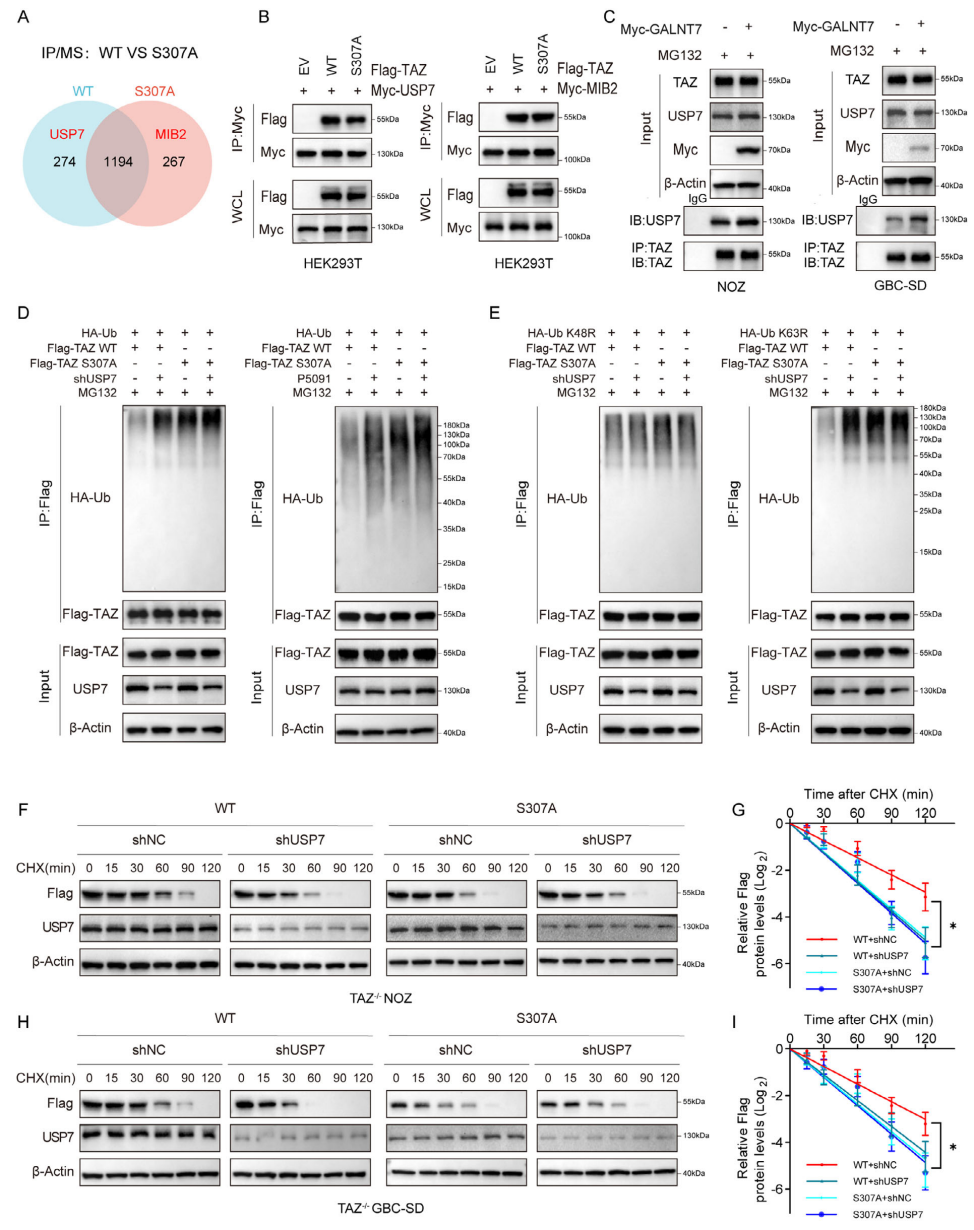
GALNT7 stabilizes TAZ by promoting USP7‐mediated TAZ deubiquitylation. (A) LC‐MS/MS of anti‐Flag immunoprecipitated from cells expressing Flag‐TAZ‐WT or Flag‐TAZ‐S307A. Venn diagram shows proteins uniquely enriched with either variant; USP7 (DUB) selectively bound TAZ‐WT, whereas MIB2 (E3 ligase) selectively bound the non‐O‐GalNAcylatable TAZ‐S307A.(B) TAZ‐S307A, which cannot be O‐GalNAcylated, showed impaired association with USP7 yet maintained MIB2 interaction, as determined by anti‐FLAG co‐IP and IB in transfected 293T cells. (C) Immunoblot analysis of NOZ and GBC‐SD cells with stably expressed Myc‐GALNT7 treated with MG132 (10 µM) for 6 h. Whole‐cell lysates (WCL) and immunoprecipitation (IP) using anti‐TAZ were used for Immunoblot analysis. (D) Depletion of USP7 or USP7 inhibitor P5091(5 µM for 24 h) increases the ubiquitination of TAZ WT, but not TAZ S307A mutant. (E) The increased TAZ ubiquitination was abolished by a K48R ubiquitin plasmid but not a K63R plasmid. (F‐I) Depletion of USP7 accelerates protein degradation (shortens half‐life) of TAZ WT, but not TAZ S307A mutant, in gallbladder cancer (GBC) cell lines. *: p < 0.05, **: p < 0.01, ***: p < 0.001; two‐way ANOVA with Tukey's test (G, I).

### 2.7 TAZ/TEAD1 Positively Regulated GALNT7 Expression at the Transcriptional Level

Unexpectedly, we observed that TAZ knockdown reduced both GALNT7 protein and mRNA, whereas ectopic TAZ up‐regulated GALNT7 expression (Figure 6A,B). This indicates that TAZ may transcriptionally regulate GALNT7. Dual ‐luciferase reporter assays confirmed TAZ transactivates the GALNT7 promoter (Figure 6C). To determine whether this transcriptional regulation depends on Ser307 O‐GalNAcylation, we examined downstream target gene expression in TAZ‐knockout cells reconstituted with wild‐type or S307A mutant TAZ. Re‐expression of TAZ‐WT significantly upregulated GALNT7, as well as canonical TAZ/TEAD targets associated with proliferation and invasion (CTGF, CYR61, ANKRD1); in contrast, the S307A mutant failed to induce these genes (Figure 6D,E). Mechanistically, nuclear/cytoplasmic fractionation revealed that GALNT7‐mediated O‐GalNAcylation promotes TAZ nuclear accumulation: overexpression of GALNT7‐WT markedly increased nuclear TAZ levels, whereas the catalytically inactive GALNT7‐Mut failed to do so (Figure 6F). Thus, O‐GalNAcylation at Ser307 stabilizes TAZ and enhances its nuclear retention, enabling transcriptional activation of target genes including GALNT7 itself. Given that TAZ functions as a transcriptional co‐activator with TEAD factors, we screened all four TEAD paralogs and identified TEAD1 as the principal regulator of GALNT7 mRNA and protein abundance (Figure 6G,H). Further dual‐luciferase assays validated TEAD1 as the key regulator (Figure 6I). Bioinformatics analysis via the JASPAR database identified three conserved TEAD1 transcription factor binding sites (TFBS)in the GALNT7 promoter (Figure 6J). Site‐directed mutagenesis of TFBS2 almost completely abrogated TEAD1‐mediated transactivation (Figure 6K). ChIP‐qPCR confirmed TEAD1 binds to TFBS2 of the GALNT7 promoter (Figure 6L). Collectively, these data establish that TAZ‐TEAD1 complexes transcriptionally up‐regulate GALNT7 through direct promoter engagement.

**FIGURE 6:**
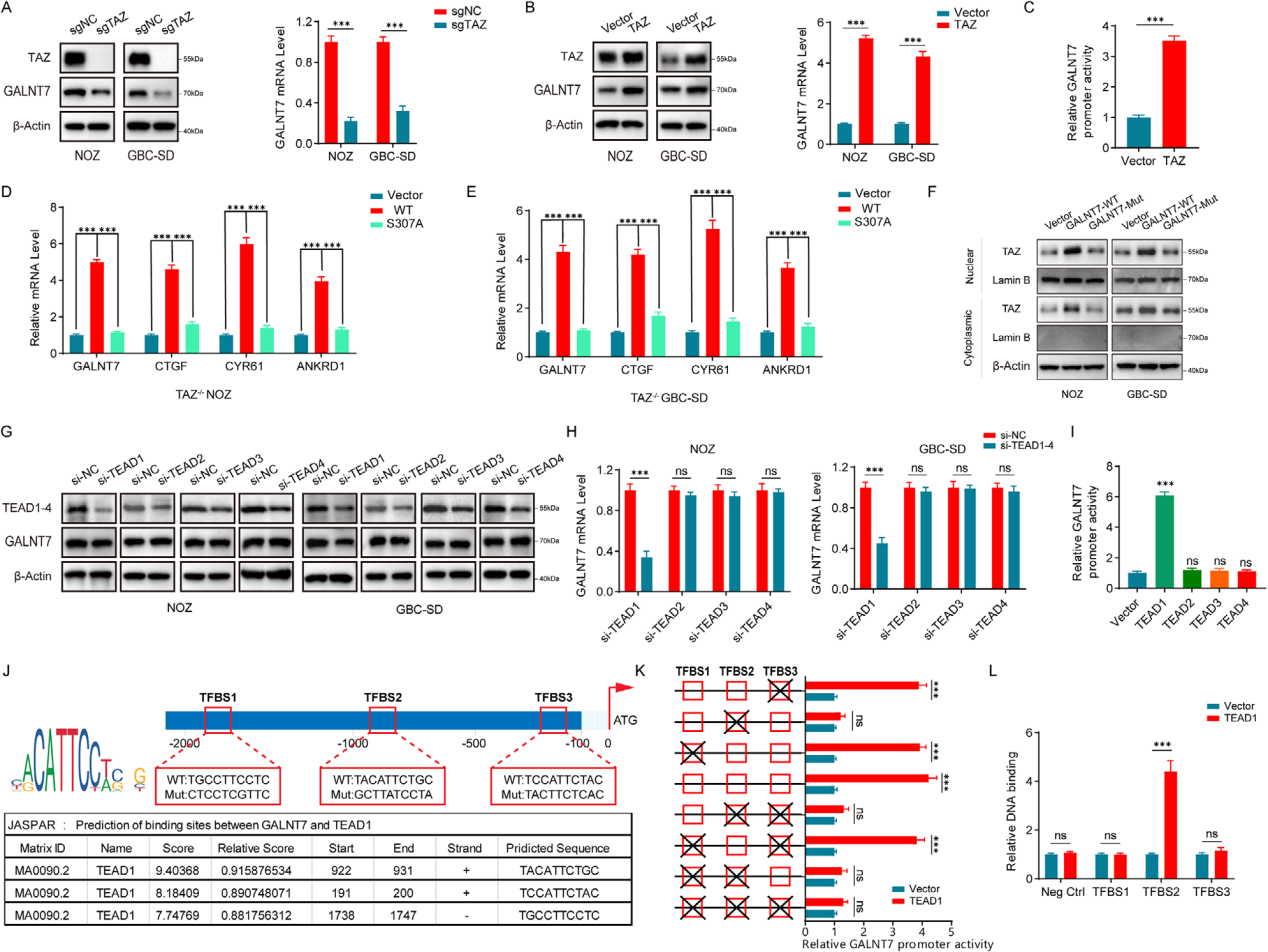
TAZ/TEAD1 positively regulated GALNT7 expression at the transcriptional level. (A) TAZ depletion reduced GALNT7 protein and mRNA levels in NOZ and GBC‐SD cells. (B) TAZ overexpression increased GALNT7 protein and mRNA levels in NOZ and GBC‐SD cells. (C) Luciferase reporter assay confirmed TAZ transactivates the GALNT7 promoter. (D‐E) qPCR analysis of TAZ‐knockout NOZ (D) and GBC‐SD (E) cells reconstituted with Vector, TAZ‐WT, or TAZ‐S307A. TAZ‐WT significantly upregulates GALNT7 and canonical TAZ/TEAD targets (CTGF, CYR61, ANKRD1), whereas S307A fails to induce these genes. (F) Nuclear/cytoplasmic fractionation showing that GALNT7‐WT increases nuclear TAZ levels, while GALNT7‐Mut does not. Lamin B and β‐Actin serve as nuclear and cytoplasmic markers, respectively. (G‐H) si‐TEAD1 reduced GALNT7 protein and mRNA levels in NOZ and GBC‐SD cells. (I) Luciferase reporter assay showing TEAD1 promoted the expression of GALNT7. (J) Schematic of the GALNT7 promoter depicting three putative TEAD1‐binding motifs (TFBS1‐3) and their mutant versions. (K) Mutagenesis revealed TFBS2 is required for TEAD1‐driven promoter activity. (L) TEAD1 specifically binds to TFBS2 within the GALNT7 promoter. ChIP‐qPCR analysis of TEAD1 occupancy at three putative TEAD1‐binding sites (TFBS1, TFBS2, TFBS3) and a distal intergenic negative control region (located ∼15 kb upstream of GALNT7, chr4:173,153,518‐173,153,670) in cells overexpressing TEAD1 or vector control. Data are represented as means ± SD in the bar graphs. *: p < 0.05, **: p < 0.01, ***: p < 0.001; two‐tailed unpaired Student's *t*‐test (A‐C, H, I, K, L); one‐way ANOVA with Tukey's test (D, E).

### 2.8 TAZ S307 O‐GalNAcylation Is Required for GALNT7‐Mediated Progression of Gallbladder Cancer

We have established that both TAZ Ser307 O‐GalNAcylation and GALNT7 overexpression drive GBC progression. However, their epistatic relationship in oncogenesis remained unresolved. To explore this, GALNT7 was stably overexpressed in TAZ‐WT and TAZ‐S307A GBC cells (Figure S6A). In vitro experiments revealed that GALNT7 could greatly increase the proliferation, migration, and invasion of TAZ WT GBC cells, but this effect was absent in TAZ S307A GBC cells. (Figure 7A–E and Figure S6B–D). In vivo research indicated that mice injected with cells expressing TAZ‐WT had larger tumors and more metastatic nodules than those injected with S307A mutant TAZ‐expressing cells. Moreover, GALNT7 overexpression further promoted tumor growth and metastasis in TAZ‐ WT expressing cells but had no effect on S307A mutant cells (Figure 7F–J). These results demonstrate that TAZ S307 O‐GalNAcylation is not only critical for the oncogenic effects of TAZ but also essential for GALNT7‐mediated progression of GBC. The inability of the S307A mutant to restore the aggressive phenotype, even with GALNT7 overexpression, underscores the pivotal role of TAZ S307 O‐GalNAcylation in driving GBC progression. These findings position TAZ S307 O‐GalNAcylation as a possible therapeutic target for treating this aggressive cancer.

**FIGURE 7:**
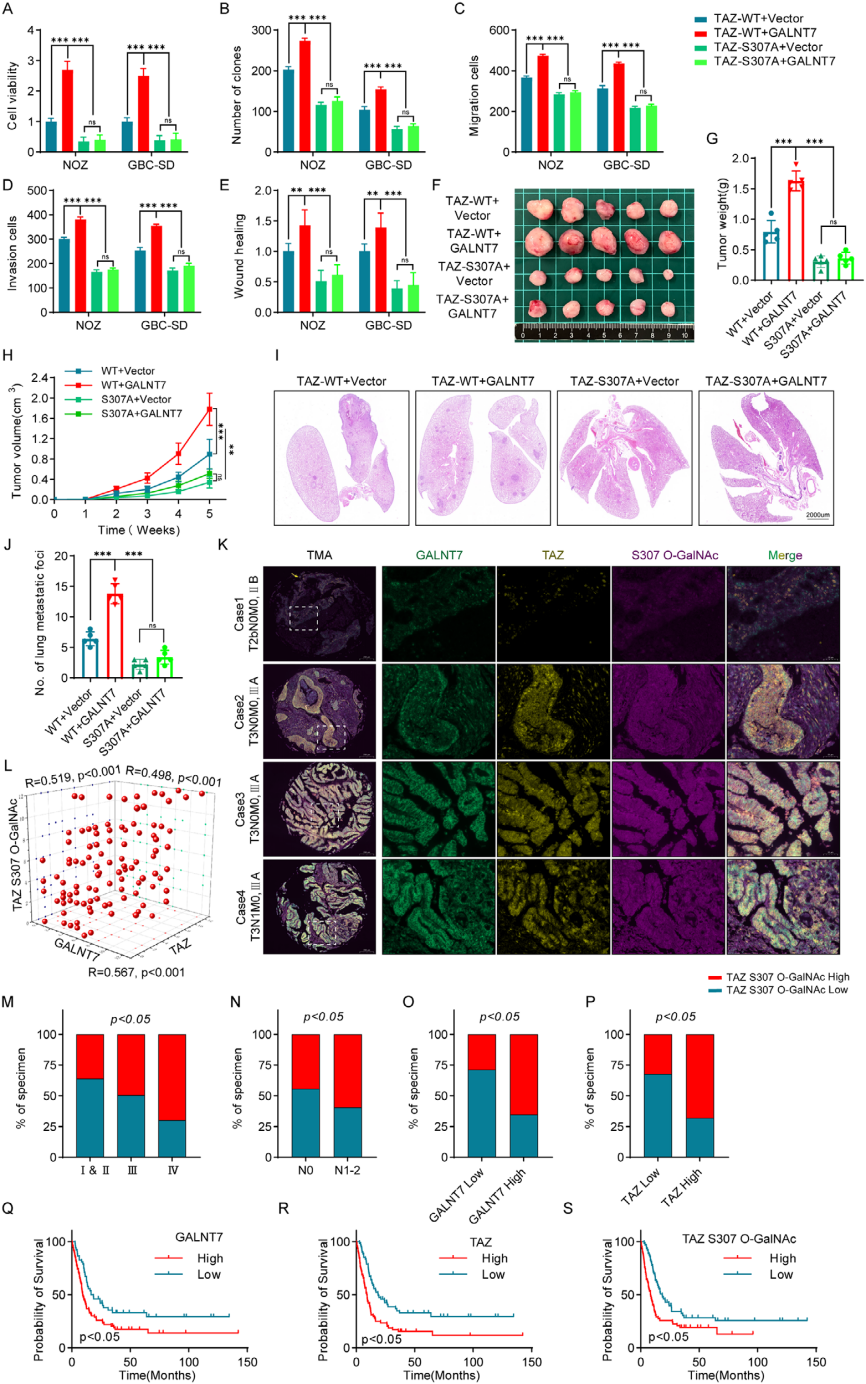
TAZ S307 O‐GalNAcylation is Required for GALNT7‐Mediated progression of gallbladder cancer. (A) CCK‐8 assay showing the proliferation of NOZ and GBC‐SD TAZ KO cells stably expressing TAZ WT or TAZ S307A and transfected with empty vector (Vector) or Myc‐GALNT7. (n = 5). (B) Quantification of Colony formation assay for the indicated cells. (n = 3). (C‐D) Migration and invasion capacities of the indicated cells were quantified by Transwell assays. (n = 3). (E) Quantification of wound healing assays for the indicated cells. (n = 3). (F) Representative photographs of subcutaneous tumors harvested 5 weeks after injection of NOZ TAZ‐KO cells expressing TAZ‐WT or TAZ‐S307A, with or without Myc‐GALNT7 co‐expression (n = 5 per group). (G‐H) Tumor weight (G) and volume (H) were quantified for each group. (I) Representative H&E staining of lung metastasis models. (n = 5 per group) (J) Quantification of lung metastatic nodules. (K) Representative immunofluorescence images of GBC tissues showing the expression of GALNT7 (green), TAZ (yellow), and TAZ S307 O‐GalNAcylation (purple) in cases with high and low levels of these markers. DNA was visualized with DAPI (blue) nuclear counterstain. Scale bar, 50 µm. (L) Scatter plot of the multiplex immunohistochemistry staining scores for GALNT7, TAZ, and TAZ S307 O‐GalNAcylation in GBC, n = 149. (M‐N) mIHC scores show TAZ‐S307 O‐GalNAcylation rising with advanced TNM (M) and N (N) stages. (O) GALNT7 protein level correlates positively with TAZ‐S307 O‐GalNAcylation. (P) TAZ abundance correlates positively with TAZ‐S307 O‐GalNAcylation. (Q‐S) Kaplan‐Meier survival curves showing the probability of survival for GBC patients with high vs. low GALNT7 expression levels (Q), TAZ expression levels (R), and TAZ S307 O‐GalNAcylation levels (S). Data are represented as means ± SD in the bar graphs. *: p < 0.05, **: p < 0.01, ***: p < 0.001; one‐way ANOVA with Tukey's test (A‐ E, G, J); two‐way ANOVA with Tukey's test (H); chi‐square test (M‐P); log‐rank test for survival analysis (Q‐S).

### 2.9 High Expression of TAZ S307 O‐GalNAcylation in Gallbladder Cancer is Associated With Worse Overall Survival

To further explore the relationship between GALNT7, TAZ, and TAZ S307 O‐GalNAcylation in GBC, we conducted multiple immunohistochemistry (mIHC) on a GBC tissue microarray containing 149 GBC patients using anti‐ GALNT7, anti‐TAZ, and anti‐ TAZ S307 O‐GalNAcylation antibodies (Figure 7K and Figure S6E). Our results revealed TAZ S307 O‐GalNAcylation levels were positively linked to GALNT7 protein levels and TAZ protein levels in various samples (Figure 7L,O,P). Besides, we analyzed the relationship between TAZ S307 O‐GalNAcylation and clinicopathological characteristics in GBC specimens, and the data indicated a positive link between the IHC score of TAZ S307 O‐GalNAcylation and the TNM and N stages in patients with GBC (Figure 7M,N). Moreover, high GALNT7, TAZ and TAZ S307 O‐GalNAcylation jointly predicted markedly shorter overall survival relative to their low‐expressing counterparts (Figure 7Q–S). To sum up, our findings indicate that GALNT7‐mediated TAZ S307 O‐GalNAcylation is a critical factor in GBC, and TAZ S307 O‐GalNAcylation could serve as a predictive marker for cancer prognosis.

### 2.10 Identification of Olaparib as a GALNT7‐mediated O‐GalNAcylation Inhibitor that Exhibits Anti‐GBC Efficacy In Vitro and In Vivo

Given GALNT7's central oncogenic function and the lack of selective inhibitors, identifying potent GALNT7 antagonists represents an urgent unmet clinical need. A virtual screening approach based on structure was utilized to dock FDA‐approved drugs to quickly discover therapeutic drugs targeting GALNT7 (Table S4). Among the ten highest‐scoring compounds, we found three with anti‐tumor activity in the PubChem database (Figure 8A). Olaparib emerged as the most promising candidate because it potently suppressed TAZ S307 O‐GalNAcylation in vitro (Figure 8B). Thus, we prioritized Olaparib for more in‐depth analysis. Our docking simulations propose that Olaparib strongly interacts with the GALNT7 protein (Figure 8C). Surface plasmon resonance assays later indicated that Olaparib and GALNT7 have a strong binding affinity (K_D_ = 4.17 µM) (Figure 8D). Dose response analysis showed that Olaparib suppressed GBC cell proliferation in a manner dependent on concentration, showing an IC50 of 6.2 µmol/L in GBC‐SD cells and 12.2 µmol/L in NOZ cells (Figure 8E). In our in vitro experiments, NOZ and GBC‐SD cells were treated with either DMSO or Olaparib and we evaluated their effects on cell proliferation, migration, and invasion. The findings indicated that Olaparib treatment significantly reduced cell growth, as shown by CCK‐8 and colony formation assays (Figure 8F,G and Figure S7A). Additionally, Olaparib markedly diminished the invasive and migratory capacity of GBC cells, as assessed by Transwell assays and wound healing assays (Figure 8H–J and Figure S7B,C).

**FIGURE 8:**
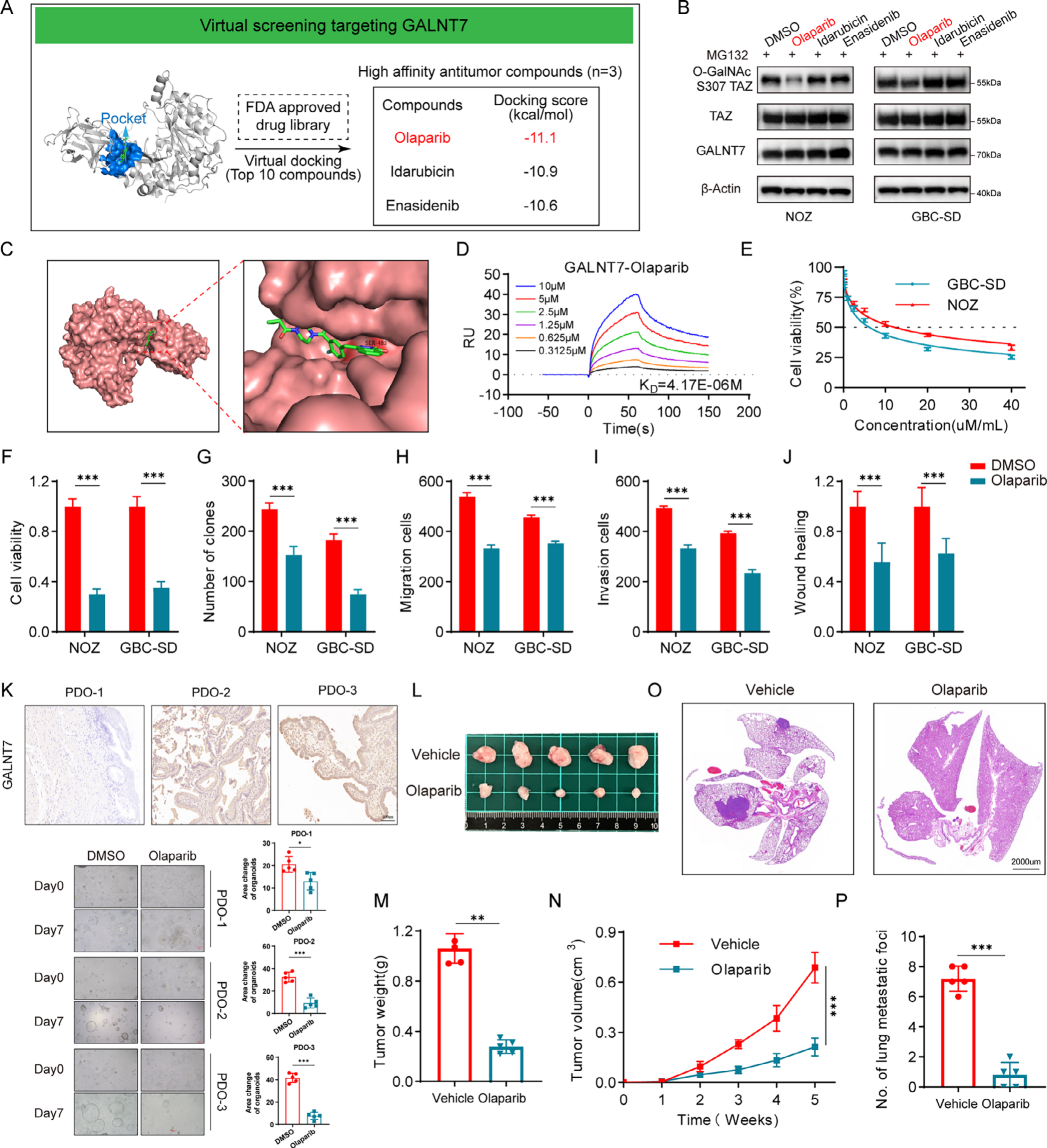
Identification of Olaparib as a GALNT7‐mediated O‐GalNAcylation inhibitor that exhibits anti‐GBC efficacy. (A) Flow diagram of GALNT7 inhibitor screening. (B) Olaparib exhibiting potent inhibitory effects on TAZ S307 O‐GalNAcylation. (C) Olaparib–GALNT7 interaction displayed in 3D binding mode. (D) Surface plasmon resonance assays depicting the binding of Olaparib to GALNT7 at increasing concentrations (0.3125 µM to 10 µM). (E) IC50 values of Olaparib in two GBC cell lines after 48 h of treatment. (F‐G) Effects of Olaparib (10 µM) on cell growth abilities of GBC cells. (n = 5 for CCK‐8 and n = 3 for colony formation assays). (H‐I) Olaparib inhibits the invasion and migration of GBC cells. (n = 3 per group). (J) Effects of Olaparib on wound healing ability of GBC cells. (n = 3). (K) Olaparib inhibits growth of patient‐derived GBC organoids with efficacy correlating with GALNT7 expression levels. (n = 3). (L‐N) Olaparib treatment significantly influences tumor growth in a subcutaneous xenograft model (n = 5 per group). (O‐P) Olaparib treatment reduces metastasis in a lung metastatic model (n = 5 per group). Data are represented as means ± SD in the bar graphs. *: p < 0.05, **: p < 0.01, ***: p < 0.001; two‐tailed unpaired Student's *t*‐test (F‐K, M, P); two‐way ANOVA with Tukey's test (N).

To rigorously establish that the observed anti‐tumor effects of Olaparib in GBC are specifically mediated through GALNT7 targeting rather than solely through its canonical PARP inhibitory activity, we utilized Niraparib—a structurally distinct PARP inhibitor that lacks GALNT7 binding affinity—as a negative control. While both Olaparib and Niraparib potently inhibit PARP, Niraparib failed to suppress TAZ S307 O‐GalNAcylation (Figure S7D) and exhibited minimal anti‐tumor effects in GBC cells, whereas Olaparib retained full efficacy. Strikingly, genetic depletion of GALNT7 in GBC cell lines completely abolished Olaparib sensitivity, with cells becoming resistant to treatment and exhibiting viability, colony formation, and migratory capacity comparable to Niraparib‐treated controls (Figure S7F–L). This strict dependency demonstrates that GALNT7 is the essential pharmacological target for Olaparib in this context, and that Olaparib does not exert significant anti‐tumor effects through GALNT7‐independent mechanisms (including alternative PARP‐mediated pathways) in GBC cells. To determine whether Olaparib exerts its anti‐tumor effects specifically through the GALNT7‐TAZ axis, we tested its efficacy in cells expressing the glycosylation‐deficient TAZ S307A mutant. CCK‐8 assays revealed that cells expressing TAZ‐S307A exhibited significantly reduced sensitivity to Olaparib compared to TAZ‐WT cells (Figure S7M), demonstrating that the GALNT7‐TAZ S307 O‐GalNAcylation axis constitutes a critical mediator of Olaparib efficacy.

Having established the essential role of GALNT7‐mediated TAZ glycosylation in Olaparib sensitivity at the cellular level, we next evaluated its therapeutic potential in clinically relevant models. We established three independent patient‐derived organoids (PDOs) from distinct GBC patients with varying baseline GALNT7 expression levels. Olaparib treatment significantly inhibited organoid growth in all three PDOs, but with markedly differential efficacy: PDO‐2 and PDO‐3 (high GALNT7) showed profound growth inhibition, whereas PDO‐1 exhibited modest but statistically significant efficacy (Figure 8K). In our in vivo experiments, we assessed the therapeutic potential of Olaparib using mouse xenograft models. Tumor sizes in mice treated with Olaparib were significantly smaller than those in the control group (Figure 8L–N). To evaluate the safety profile of Olaparib in vivo, we monitored body weight changes and major organ pathology in nude mice bearing subcutaneous xenografts. Following 4 weeks of treatment with Olaparib at 50 mg/kg/day, mice showed no significant difference in body weight compared to the vehicle control group (Figure S7N). Furthermore, H&E staining of major organs including heart, liver, and kidney revealed no apparent pathological damage (Figure S7O). These findings indicate that Olaparib exhibits favorable in vivo tolerability at this dosage, without causing significant systemic toxicity. Additionally, Olaparib treatment reduced the number of metastatic nodules in the lungs, demonstrating its potential to inhibit metastasis (Figure 8O,P). Thus, Olaparib represents a clinically actionable GALNT7 antagonist with potent anti‐tumor and anti‐metastatic activity in GBC.

## 3 Discussion

Genomic profiling of gallbladder cancer (GBC) has identified recurrent mutations in TP53, KRAS, and ERBB3 [26]; however, how post‐translational modifications (PTMs)—especially glycosylation—drive disease progression remains largely unexplored. Our study positions O‐GalNAcylation as a central regulator of GBC aggressiveness. We demonstrate that GALNT7 glycosylates TAZ at Ser307, thereby blocking K48‐linked ubiquitination and unleashing TEAD1‐dependent transcription. Notably, we rigorously excluded O‐GlcNAcylation as a confounding modification; despite OGT being upregulated in GBC proteomics (Figure 1B), TAZ does not undergo O‐GlcNAcylation at Ser307 or elsewhere, as confirmed by Co‐IP and Anti‐O‐GlcNAcylation antibody blotting (Figure S4D), establishing O‐GalNAcylation as the specific regulatory PTM on TAZ. Unlike the ligand–receptor‐centric GALNT6‐MUC1 axis in mammary carcinogenesis [27] or the GALNT1–CD44 circuit in gastric cancer [19], the GALNT7–TAZ pathway bypasses membrane receptors and directly hijacks a transcriptional co‐activator, establishing a self‐amplifying oncogenic loop. This reveals that individual GALNT isoenzymes can orchestrate fundamentally different oncogenic logics across tissues.

Previous studies have pointed out the crucial role of GALNT7 in cancer progression [28, 29]. GALNT7 functions as a pivotal regulator of cell‐surface glycosylation and glycocalyx architecture, with its O‐glycosylation activity poised at the nexus of cellular signaling networks [30]. By sculpting the glycan landscape, this lone gene can orchestrate oncogenic circuits that propel tumor evolution. Consistent with these finding, our study reveals that GALNT7 is highly expressed in GBC tissues and correlates with unfavorable clinical results, including liver metastasis and reduced overall survival, substantiating GALNT7's oncogenic properties. Silencing GALNT7 greatly reduced cell proliferation and invasion in GBC cells. Similarly, GALNT7 silencing significantly hindered tumor development in a xenograft model and lung metastasis in mouse models. Thus, GALNT7 might be a possible predictive biomarker and therapeutic target for GBC.

TAZ, the transcriptional co‐activator paralog of YAP, is the central on/off switch of the Hippo pathway: when kinases LATS1/2 are active, TAZ is phosphorylated on four canonical serines, sequestered in the cytoplasm and tagged with K48‐linked ubiquitin for proteasomal destruction; when the Hippo pathway is not active, TAZ translocates to the nucleus, partners with TEAD factors and drives proliferation‐survival genes [31]. Against this backdrop, we reveal that the same residue cluster is subject to an additional, glycosylation‐based control. The mechanistic link between GALNT7 and TAZ represents a significant discovery. By stabilizing TAZ through O‐GalNAcylation at Serine 307, GALNT7 prevents its proteasomal degradation, thereby enhancing TAZ‐mediated oncogenic signaling. Critically, this stabilization strictly requires GALNT7's glycosyltransferase catalytic activity; the catalytically dead mutant (GALNT7‐Mut, with D301N/H303D/E305Q substitutions) completely failed to stabilize TAZ or promote GBC progression (Figure S3), formally excluding a mere scaffold mechanism and establishing enzymatic dependency. While GALNT7 predominantly resides in the Golgi apparatus (co‐localizing with GM130) and TAZ shuttles between cytoplasm and nucleus, high‐resolution confocal microscopy revealed partial co‐localization of TAZ with GM130 and GALNT7 within the Golgi (Figure 3E, Figure S2C). This suggests that TAZ may associate with Golgi membranes/cisternae or be captured during vesicular trafficking through the Golgi apparatus to encounter GALNT7. Notably, this O‐GalNAcylation acts independently of canonical Hippo pathway regulation; GALNT7 overexpression did not alter p‐TAZ Ser89 levels in either TAZ‐WT or TAZ‐S307A cells (Figure S4G), indicating that O‐GalNAcylation stabilizes TAZ without interfering with LATS1/2‐mediated phosphorylation or creating spatial hindrance for the phosphorylation machinery. This post‐translational modification adds a novel layer to the known regulatory mechanisms of TAZ. Using high‐resolution IP‐MS and a newly generated anti‐TAZ S307 O‐GalNAcylation antibody, we map the exact sugar acceptor residue and demonstrate stoichiometric occupancy in patient tumors. Lectin blotting revealed that TAZ carries exclusively the simple Tn antigen (GalNAcα1‐Ser/Thr) at Ser307 without further elongation to sialyl‐Tn (SNA‐negative) or T antigen (PNA‐negative; Figure 4C, Figure S4A,E), suggesting a discrete recognition motif for USP7 recruitment rather than complex glycan processing. This positions O‐GalNAcylation as a master PTM controlling transcriptional co‐activator fate. Besides, we show that S307 O‐GalNAcylation does not merely sterically hinder the proteasome; rather, it remodels the TAZ surface to recruit the deubiquitinating enzyme USP7, leading to precise removal of K48‐linked ubiquitin chains—a case of “PTM editing” that shifts the E3/DUB balance. Notably, while this study establishes glycosylation as a regulator of TAZ stability, we did not investigate potential interplay between phosphorylation and O‐GalNAcylation, leaving this question for future work.

Traditionally, GALNT enzymes are thought to initiate mucin‐type O‐glycosylation, modifying membrane‐bound or secreted mucins to potentiate EGFR or β‐catenin pathways [9, 32, 33]. Our data reveal that TAZ, a nuclear co‐activator, is an equally efficient substrate. The Ser307 motif is evolutionarily conserved (Figure S4C) yet lacks mucin‐like repeats, suggesting a hidden glyco‐code within transcriptional regulators. This substrate promiscuity challenges the mucin‐centric dogma and argues for systematic glycoproteomic screens to map the “non‐canonical” GALNTome across cancers. Although proteomics revealed altered expression of other glycosyltransferases (Figure 1B), the strict enzymatic dependency of Ser307 modification—evidenced by complete loss upon GALNT7 knockdown and failure of GALNT7‐Mut (Figure 4G, Figure S3)—supports GALNT7‐specific targeting, though potential compensation by other GALNTs upon chronic inhibition remains uncharacterized.

The positive feedback loop between GALNT7 and TAZ, mediated by TEAD1, further underscores their combined effect in driving GBC progression. Cell fractionation confirmed that GALNT7 markedly increases transcriptionally active nuclear TAZ pools (Figure 6F), providing the mechanistic basis for subsequent TEAD1‐mediated transcriptional upregulation of GALNT7. Because GALNT7 itself is transcriptionally up‐regulated by TAZ/TEAD1, a self‐reinforcing feed‐forward loop is created that locks TAZ in a chronically stable state. Such glyco‐deubiquitin “double‐lock” circuitry has not previously been described for any oncoprotein and may constitute a general paradigm for GALNT‐family enzymes in solid tumors. This interplay may explain the aggressive nature of GBC and offers a rationale for targeting this axis therapeutically.

Selective GALNT7 inhibitors are unavailable [34]. By structure‐based docking, we identified that Olaparib — an FDA‐approved PARP inhibitor with a well‐established safety profile — can be repurposed as a potent GALNT7 antagonist represents a rare and immediately actionable “bench‐to‐bedside” opportunity [35]. Using structure‐guided docking and SPR, we unveil that Olaparib occupies the pocket of GALNT7 and competitively suppresses TAZ O‐GalNAcylation. We validated GALNT7‐specificity over PARP inhibition: Niraparib (structurally distinct PARP inhibitor lacking GALNT7 binding) failed to suppress TAZ O‐GalNAcylation, whereas GALNT7 depletion abolished Olaparib sensitivity (Figure S7D–L). Furthermore, TAZ‐S307A cells showed reduced Olaparib sensitivity (Figure S7M), confirming the GALNT7‐TAZ axis as the pharmacological target. Notably, Olaparib binds GALNT7 with micromolar affinity. Despite this relatively modest binding affinity, pharmacodynamic studies demonstrate that oral administration at 50 mg/kg/day achieves sufficient tissue exposure to markedly suppress TAZ S307 O‐GalNAcylation and exert potent anti‐tumor effects (Figure 8N–P). Importantly, this dosing regimen showed no significant systemic toxicity, as evidenced by stable body weights and normal histology of major organs (Figure S7N–O). Thus, clinically achievable and well‐tolerated concentrations of Olaparib are sufficient for effective GALNT7 inhibition in vivo, supporting a favorable therapeutic index for GBC treatment. In patient‐derived organoids Olaparib shows anti‐tumor effects, indicating a therapeutic index independent of PARP inhibition. These data enable immediate investigator‐initiated basket trials in GALNT7‐high GBC, circumventing de‐novo drug development timelines.

Our study delineates the GALNT7‐TAZ axis in GBC, yet several constraints temper its conclusions. The retrospective, single‐center tissue cohort narrows prognostic generalizability, and the absence of high‐resolution structural data obscures how S307 O‐GalNAcylation sculpts TAZ–USP7 contacts. Current animal experiments were limited to subcutaneous xenografts and tail‐vein metastasis models in immunocompromised mice, lacking orthotopic microenvironment and immune context. While PDO studies partially address this (Figure 8K), the scarcity of validated GBC models precluded PDX or orthotopic validation.

## 4 Conclusion

Our study reveals the GALNT7‐TAZ axis as a significant contributor to GBC progression and emphasizes its potential for therapeutic intervention. By elucidating the molecular mechanisms underlying this axis, we provide a foundation for developing novel strategies to improve outcomes for GBC patients.

## 5 Experimental Section

### 5.1 Ethics Approval

GBC tissues along with normal gallbladder tissues were surgically obtained from Tongji Hospital (Wuhan, China), with approval from the Ethics Committee (TJ‐IRB202412108). All patients provided written authorization for tissue usage. The protocol adhered to the Helsinki Declaration and gained ethical endorsement from Tongji Hospital's Human Research Ethics Committee at Huazhong University of Science and Technology.

### 5.2 4D‐Label‐Free Quantitative Proteomics

Five paired GBC and adjacent non‐tumor tissues were lysed in SDT buffer (4%SDS, 100 mM DTT, 150 mM Tris‐HCl pH 8.0). Protein (200 µg) was processed by FASP: depletion with 8 M urea/150 mM Tris‐HCl pH 8.0 (Microcon‐10 kDa), alkylation with 100 mM IAA, and overnight digestion with 4 µg trypsin (Promega) at 37°C. Peptides were desalted (C18), dried, and re‐dissolved in 0.1% FA. LC was performed on an Easy‐Spray C18 column (75 µm × 10 cm, 3 µm) using a 0–35% B gradient (300nL/min). A timsTOF Pro operated in PASEF mode (m/z 100–1700; 1/k_0_ 0.6–1.6; 10 frames per cycle).MS data were searched with MaxQuant 1.5.3.17 against UniProt_human (release 2023‐02, 20596 entries) using 6 ppm/20 ppm tolerance, carbamidomethyl (C) as fixed, oxidation (M) as variable, ≤ 2 missed cleavages, FDR ≤ 1% (peptide and protein level). LFQ intensities (min ratio count = 1) were used for quantification. All proteomic experiments were performed at Shanghai Applied Protein Technology (Shanghai, China).

### 5.3 Tissue Microarray (TMA) and Multiplexed Immunohistochemistry (mIHC)

A tissue microarray was assembled by Shanghai YBL Biotechnology (Ethics approval YBL202411205). Multiplex immunohistochemistry followed the YBL‐mIHC032 protocol supplied with the kit. Primary antibodies were applied sequentially: GALNT7 (TYR‐520, green), TAZ (TYR‐570, yellow), and TAZ S307 O‐GalNAcylation (TYR‐620, purple); nuclei were counter‐stained with DAPI. Slides were imaged on a multi‐channel fluorescence scanner. Staining intensity (0–3) and positive‐cell percentage (0–4 scale) were multiplied to yield an overall score (0–12). Tumors with ≤4 points were classed as low expression; >4 as high.

### 5.4 Generation of GALNT7 Catalytic Mutant

GALNT7 catalytic mutant (GALNT7‐Mut) was generated by substituting three amino acid residues in the glycosyltransferase 1 (GT1) motif, which were previously identified to be critical for enzymatic activity of murine GALNT1 (ppGalNAc‐T1) and conserved among all 20 human GALNT family members. Based on the structure‐function analysis by Hagen et al. [36]. The substitutions include Asp301→Asn (corresponding to Asp209 in murine GALNT1), His303→Asp (corresponding to His211 in murine GALNT1), and Glu305→Gln (corresponding to Glu213 in murine GALNT1).

### 5.5 Lectin Pull‐Down Assay

Total protein was isolated using a Beyotime extraction kit. Glycoprotein complexes were enriched by incubating lysates with agarose‐conjugated Vicia villosa lectin (Vector Labs) for 2 h at 4°C, followed by washing, elution, and Western blot analysis.

### 5.6 Surface Plasmon Resonance (SPR) Measurement

The binding of GALNT7 to Olaparib was evaluated on a Biacore 1K system (Cytiva, Sweden) equipped with a CM5 sensor chip. GALNT7 (50 µg/mL, pH 4.0) was injected over the active flow cell at 10 µL/min for 5 min, followed by blocking with 1 M ethanolamine (10 µL/min, 10 min). A reference cell was prepared in parallel using pH‐adjusted PBS without protein. Olaparib solutions (0–50 µM in PBS + 5% DMSO) were flowed at 30 µL/min for 60 s association and 90 s dissociation. Kinetic analysis was performed with Biacore Insight v4.0.8.20368 to obtain the K_D_ value.

### 5.7 Virtual Screening and Molecular Docking Analysis

To pinpoint potential GALNT7 inhibitors, we screened the FDA National Drug Code Directory. Filters were applied: MW 108–508, complexity 100–500, ≤10 H‐bond acceptors, ≤5 donors (Lipinski‐compliant). Low‐energy 3‐D conformers were generated with RDKit and minimized using the MMFF force field. The GALNT7 structure was predicted with AlphaFold. Docking was executed in AutoDock Vina (grid center 18.8, 192.1, 1.4; grid size 18.4, 23.1, 22.2). The ten highest‐ranking poses were visualized with PyMOL.

### 5.8 In Vivo Experiments

Animal maintenance and experimental procedures adhered to the NIH Guidelines for the Care and Use of Laboratory Animals and were approved by the Tongji Hospital Ethics Committee (HUST, Wuhan, China) (permit number: TJH‐202409014). For xenograft studies, NOZ cells (5 × 10^6^) were injected subcutaneously into the flanks of 5‐week‐old nude mice (n = 5 per group). Tumor volume was measured every week using calipers, and tumor size was calculated as (length × width^2^)/2. For metastasis assays, cells (2 × 10^6^) were injected into the tail vein, and lung metastases were assessed after 6–8 weeks by H&E staining of lung sections. Mice were euthanized following institutional guidelines, and tissues were collected for further analysis. To evaluate the effect of Olaparib in animal models, Olaparib was pre‐dissolved in DMSO to prepare a 312.5 mg/ml mater liquid, then the stock liquid was sequentially diluted with PEG300 (MedChemExpress, HY‐Y0873) and PBS. Mice were orally administered Olaparib at a dose of 50 mg/kg/day. The detailed animal experimental data are provided in Table S10.

### 5.9 Statistical Analysis

All analyses were conducted with GraphPad Prism 9.0. A minimum of three independent experiments were performed for each assay to ensure statistical reliability. Data are reported as mean ± standard deviation (SD). Comparisons between two groups were evaluated with student's two‐tailed *t*‐test; while one‐way ANOVA with Tukey's test, or two‐way ANOVA with Tukey's test was used for multiple groups. Categorical variables were assessed by the chi‐square test, and correlations were analyzed with Spearman's rank test. Survival curves were constructed using Kaplan‐Meier estimates and compared via log‐rank tests. Statistical significance was defined as *p < 0.05, ** p < 0.01, and *** p < 0.001; p ≥ 0.05 was considered not significant.

## Author Contributions

**Peng Qiu**, **Zhengdong Deng**, **Ming Zhang** and **Yunxiang Feng** conceived the idea and designed the project, performed most of the experiments, analyzed data, and drafted the manuscript. **Yibo Deng** acquired the clinical data, performed statistical analysis, and revised the manuscript. **Kai Zhao**, **Xiangyu Li**, **Yun Lu**, **Li Tian**, **Tao Yang**, **Wei Yao** provided help for functional experiments. **Jianming Wang** and Zhengdong Deng supervised the entire project. All authors read and approved the final committed version of the manuscript.

## Funding

The study was supported by the National Natural Science Foundation of China (grant no. 82403218 and grant no. 82373032) and Central Guide to Local Science and Technology Development Project of Hubei Province (grant no. 2024EIA009).

## Ethics Approval and Consent to Participate

GBC tissues along with normal gallbladder tissues were surgically obtained from Tongji Hospital (Wuhan, China), with approval from the Ethics Committee (TJ‐IRB202412108). All patients provided written authorization for tissue usage. The protocol adhered to the Helsinki Declaration and gained ethical endorsement from Tongji Hospital's Human Research Ethics Committee at Huazhong University of Science and Technology. Animal maintenance and experimental procedures adhered to the NIH Guidelines for the Care and Use of Laboratory Animals and were approved by the Tongji Hospital Ethics Committee (HUST, Wuhan, China) (permit number: TJH‐202409014).

## Conflicts of Interest

The authors declare no potential conflicts of interest.

## Supporting information




**Supporting file 1**: advs76490‐sup‐0001‐SuppMat.docx.

**Supporting file 2**: advs76490‐sup‐0002‐Table S1.xlsx.

**Supporting file 3**: advs76490‐sup‐0003‐Table S2.xlsx.

**Supporting file 4**: advs76490‐sup‐0004‐Table S3.xlsx.

**Supporting file 5**: advs76490‐sup‐0005‐Table S4.xlsx.

**Supporting file 6**: advs76490‐sup‐0006‐Table S10.xlsx.

## Data Availability

APTBIO performed the 4D label‐free proteomics; quantitative values are in Supplementary Table S1, and further data can be requested from the corresponding authors.
