# Supplementary material for: Targeting GALNT7 Disrupts the TAZ O‐GalNAcylation Feedback Loop to Suppress Gallbladder Cancer Progression
Source: Adv Sci (Weinh). 2026 Jul 11:e76490. Online ahead of print. doi: 10.1002/advs.76490 (PMC13355891; doi:10.1002/advs.76490)
Supplement: Supplementary file 1 — Supporting file 1: advs76490‐sup‐0001‐SuppMat.docx. [file ADVS-9999-e76490-s006.docx]

**Supplementary Materials and methods**

**Cell Culture**

NOZ cells ( RRID: CVCL_3079) were obtained from the Health Science Research Resources Bank, Osaka, Japan. GBC-SD cells ( RRID: CVCL_6903) were obtained from the Chinese Academy of Sciences, Shanghai. HEK293T cells ( RRID: CVCL_0063) were obtained from the American Type Culture Collection (ATCC). All cell lines were obtained from commercial repositories, not derived in-house. Cells were maintained in DMEM (Gibco, USA) supplemented with 10% fetal bovine serum (Gibco, USA) and 100 µg/mL streptomycin and 100 U/mL penicillin (Gibco, USA) at 37 °C with 5% CO2. NOZ cells between passages 5 and 15, GBC-SD cells between passages 3 and 12, and HEK293T cells between passages 4 and 10 were used for all experiments. Cell line authentication (STR profiling) was performed before and after the study, and all lines tested negative for mycoplasma contamination. The detailed authentication and mycoplasma detection data are provided in [Raw data.rar](https://submission.wiley.com/api/rex/v2/submissionupload/tenant/1/submission/2a3c9dd0-0162-4bcb-812e-b3f1c25d95cf/content/209bfb73-c079-45c5-9fcc-bca56a492c6f/download).

**Plasmid Construction,** **Transfection, and Lentiviral Packaging**

GALNT7, TAZ and TEAD1-4 cDNA fragments were amplified by PCR and cloned into appropriate expression vectors (e.g., pLVX, pCDH) using standard molecular cloning techniques. Point-mutated versions (e.g., TAZ-S307A) were introduced with the QuikChange site-directed mutagenesis kit (Agilent). Short-hairpin RNA sequences were designed in-house, synthesized by Sangon Biotech and ligated into pLKO.1-puro (Table S7). All final constructs were verified by Sanger sequencing. For virus production, HEK293T cells were co-transfected with the transfer plasmid plus psPAX2 and pMD2.G using PEI (Yeasen). Medium was refreshed after 6 h; viral supernatants were harvested 72 h later, filtered (0.45 µm) and applied to target cells in the presence of 8 µg/ml polybrene (Yeasen) for 8 h. Stable pools were selected with the corresponding antibiotics for ≥7 days beginning 48 h post-infection.

**Construction of TAZ-knockout cell lines by CRISPR/Cas9 gene editing**

A 20-nt guide (5′-CTGCTGGTGGAAACCCGGCAG-3′) targeting exon 2 of TAZ was selected with Benchling and cloned into BbsI-digested PX459 (Addgene #48139). GBC cells were transfected with the resulting sgRNA/Cas9 plasmid, exposed to 1 µg/ml puromycin 48 h later for 72 h, and then single-cell cloned by limiting dilution. Indels were screened by Sanger sequencing (Tsingke), and loss of TAZ protein was confirmed by immunoblot.

**CCK-8 Assay and IC50 measurements**
Proliferation was quantified with Cell-Counting Kit-8 (Dojindo). Cells (5 × 10³ per well) were plated in 96-well plates; 10 µl CCK-8 reagent was added at 24, 48 or 72 h and incubated for 2 h before absorbance reading at 450 nm (Bio-Rad microplate reader). Drug-response curves were generated by serial dilution and used to calculate half-maximal inhibitory concentrations.

**Colony Formation Assay**

A total of 1 × 10³ cells were seeded in 6-well plates and cultivated for 10–14 days. Colonies were fixed with 4% paraformaldehyde, stained with 0.1% crystal violet and counted manually (≥50 cells per colony).

**Transwell Assay**

For invasion assays, Transwell inserts (8 µm, Corning) were pre-coated with Matrigel (BD Biosciences); migration assays omitted the Matrigel layer. Serum-starved cells (5 × 10⁴ in 200 µl) were loaded into the upper chamber, and 700 µl DMEM + 10% FBS was placed below. After 24–48 h, non-invading cells were scraped away; those on the underside were fixed, stained with crystal violet and enumerated under a light microscope.

**Wound Healing Assay**
Confluent monolayers in 6-well plates were scratched with a sterile 200 µl pipette tip, washed twice with PBS and cultured in serum-free medium to limit proliferation. Images were captured at 0, 24 and 48 h (Nikon phase-contrast). Scratch width was measured in ImageJ and closure percentage calculated as (initial scratch width − final scratch width)/initial scratch width × 100%.

**GBC organoid culture**

Minced gallbladder carcinoma specimens were enzymatically dissociated in PBS (Gibco) containing type IV collagenase at 37 °C for 30–60 min. The suspension was sequentially passed through a 100μm cell strainer and centrifuged to pellet the epithelial-rich fraction. Cells were resuspended in ice-cold organoid medium, mixed 1:2 with Mogengel (Xiamen Biotecnology, China), and plated as 30-μL domes in 24-well plates. After polymerization at 37°C, 500μL complete organoid medium was overlaid, and cultures were maintained at 37 °C with 5 % CO₂ in a humidified incubator.

**Dual Luciferase Reporter Assay**

The GALNT7 promoter was amplified and ligated into pGL4.10-luc (Promega). Cells were co-transfected with pGL4.10-promoter, pRL-TK (Renilla) and experimental plasmids using Lipofectamine 3000. Firefly and Renilla activities were quantified 48 h later with the Dual-Luciferase system (Promega); Firefly values were normalized to Renilla.

**Western Blotting**

Whole-cell lysates were prepared in RIPA buffer containing protease and phosphatase inhibitors (Roche). Protein concentrations were determined with the BCA kit (Thermo Fisher). Equal amounts were resolved by SDS-PAGE, transferred to PVDF membranes (Millipore), blocked with 5% non-fat milk and probed overnight at 4 °C with primary antibodies. After HRP-conjugated secondary incubation, bands were visualized with ECL (Thermo) and quantified by ImageJ. Antibody details are given in Table S8. Original Western blot images are available in

[Raw data.rar](https://submission.wiley.com/api/rex/v2/submissionupload/tenant/1/submission/2a3c9dd0-0162-4bcb-812e-b3f1c25d95cf/content/209bfb73-c079-45c5-9fcc-bca56a492c6f/download).

**Histology and** **Immunohistochemistry**

Tissues were fixed in 10% neutral-buffered formalin, paraffin-embedded and sectioned (4 µm). H&E staining was used for morphology. For IHC, antigen retrieval was followed by 3% H₂O₂ quenching and 5% BSA blocking. Primary antibodies were applied overnight at 4 °C; HRP-conjugated secondaries were used for detection. Staining intensity (0–3) and positive-cell percentage (0–4 scale) were multiplied to yield an overall score (0–12). Tumours with ≤4 points were classed as low expression; >4 as high. Two pathologists scored each core blinded, and the mean value was recorded.

**Co-Immunoprecipitation and IP-MS**

Cells were lysed in 20 mM Tris-HCl pH 7.4, 150 mM NaCl, 1 mM EDTA, 1% NP-40 plus inhibitors. Lysates were cleared by centrifugation, incubated overnight at 4 °C with the indicated antibody and Protein A/G magnetic beads (Thermo). Beads were extensively washed, and bound proteins were eluted with 0.1 M citric acid (pH 3.0) and neutralised. For MS, eluates were separated briefly on SDS-PAGE, stained with Coomassie (Dingguo), excised, trypsin-digested and analysed on a Q Exactive HF-X coupled to an RIGOL L-3000 nano-LC. Raw files were searched against the human UniProt database with Proteome Discoverer 2.4.

**GST Pull-Down Assay**

Bacterial lysates expressing the GST-tagged bait were prepared in ice-cold GST lysis buffer (50 mM Tris-HCl pH 7.4, 150 mM NaCl, 1 mM EDTA, 0.5% NP-40, protease-inhibitor cocktail). Cleared lysates were rotated with GST magnetic beads at 4 °C overnight. After three washes, bead-bound proteins were eluted by boiling in 2× SDS loading buffer for 15 min and immediately analysed by immunoblotting.

**Immunofluorescence Staining**

Cells on coverslips were fixed with 4% paraformaldehyde, permeabilised with 0.2% Triton X-100, blocked with 5% BSA and incubated overnight at 4 °C with primary antibodies. Alexa-Fluor secondaries (Invitrogen) and DAPI were applied before imaging with a Nikon fluorescence microscope.

**Total RNA Isolation and quantitative real-time PCR(RT-qPCR)**

Total RNA was isolated with TRIzol (Vazyme) and reverse-transcribed using HiScript III RT SuperMix. qPCR was performed with ChamQ SYBR Master Mix on a CFX96 (Bio-Rad). β-Actin served as endogenous control; fold changes were calculated by 2⁻ΔΔCt.

**Nuclear/Cytoplasmic Fractionation**

Fractionation was performed using the Beyotime Nuclear and Cytoplasmic Protein Extraction Kit (P0027). Cells were lysed in buffer A (with 1 mM PMSF) on ice for 15 min, followed by buffer B and centrifugation (14,000g, 5 min, 4°C) to collect cytoplasmic supernatant. Nuclear pellets were extracted in nuclear lysis buffer with periodic vortexing (14,000g, 10 min, 4°C). Lamin B1 and β-actin served as loading controls.

**ChIP-quantitative PCR**

Cells were cross-linked with 1% formaldehyde, quenched with 125 mM glycine, lysed and sonicated to 200–800 bp fragments. Chromatin was immunoprecipitated with specific antibodies and Protein A/G beads. After washing and elution, cross-links were reversed, DNA purified and analysed by qPCR with primers listed in Table S9.

**Supplementary Figures and Table legends**

**Figure S1. GALNT7 promotes the proliferation, invasion, and migration of GBC.** (A) Colony formation assays to examine the proliferation of GALNT7 knockdown and overexpression of stably transfected NOZ and GBC-SD cell lines. (B) Cell migration and invasion assay of GALNT7 knockdown or overexpression and control cells. (C) Wound healing assays demonstrate accelerated wound closure in GALNT7 overexpressing cells and delayed closure in knockdown cells.


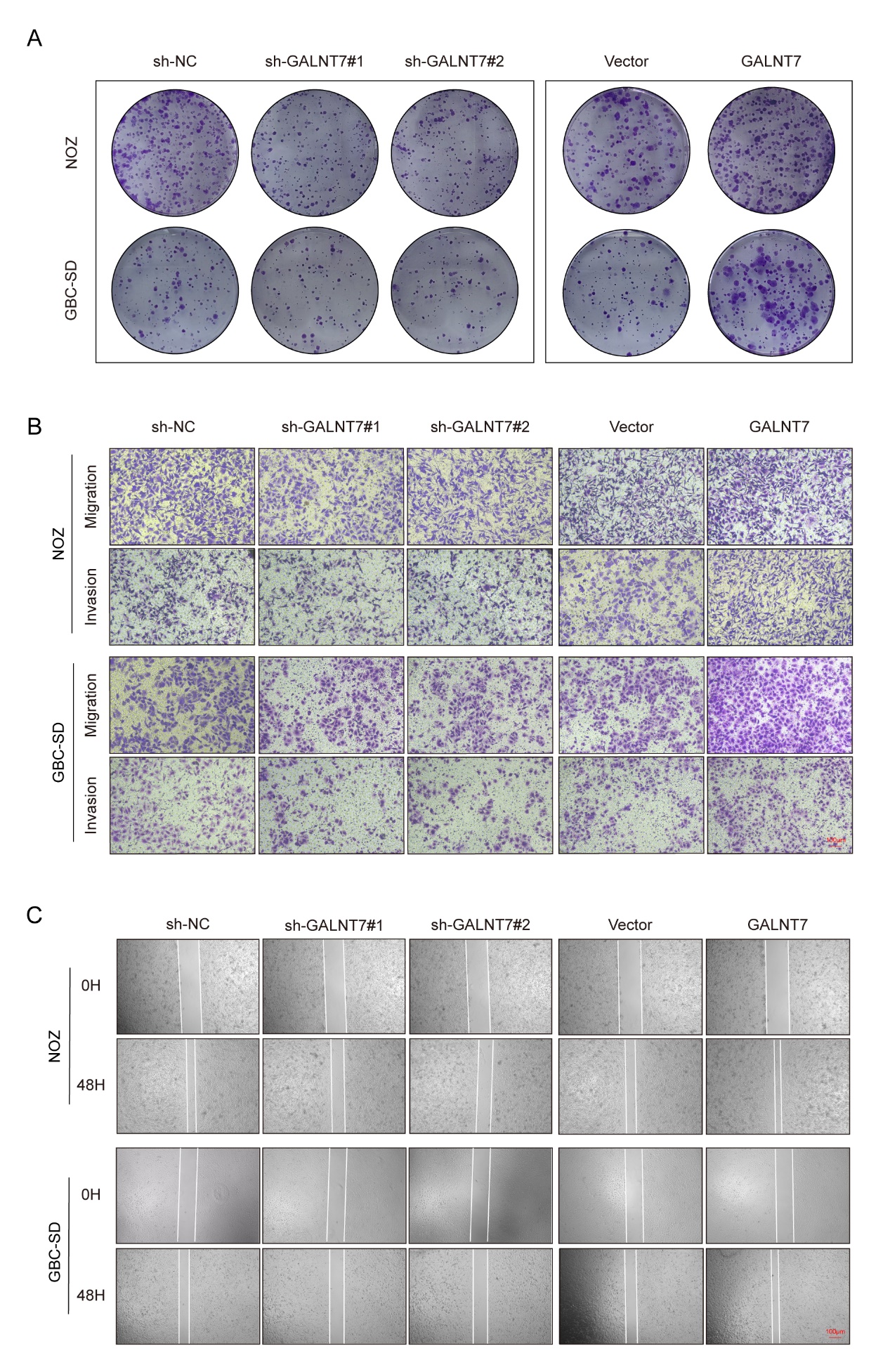


**Figure S2. GALNT7 physically interacts with TAZ and blocks its proteasomal degradation.** (A) The spectrograms showed mass spectroscopy-identified TAZ peptides of GALNT7 interacting proteins in NOZ cells. (B) HEK293T cells transfected with Myc-GALNT7 and Flag-TAZ showed reciprocal pull-down of the two proteins. (C) High-resolution confocal microscopy reveals Golgi-localized GALNT7 partially colocalizes with TAZ. Representative confocal images of endogenous GALNT7 (green), TAZ (red), GM130 (magenta, cis-Golgi marker), and DAPI (blue, nucleus) in NOZ cells. Scale bar, 20 µm. (D) Multiplex IHC of a GBC tissue microarray showed GALNT7 (green) and TAZ (yellow) colocalization; line profiles quantify overlap. Scale bar, 50 µm. (E) Co-IP mapping with serial truncations of TAZ and GALNT7 identified the mutual binding domains. (F) NOZ and GBC-SD cells with stably knockdown GALNT7 were treated with or without 3-MA (5 mM) for 6 h. Immunoblotting analysis was used to examine GALNT7 and TAZ expression. (G) The degradation rate of TAZ protein in GBC-SD cells was examined following treatment with shGALNT7 or shNC. Cells were treated with cycloheximide (CHX, 50 µM) to inhibit protein synthesis, and TAZ protein levels were measured at various time points (0, 30, 60, 90, and 120 minutes) after CHX treatment. The relative protein levels are expressed as log2 fold change normalized to the internal reference β-Actin and the protein expression at the t = 0 time point. (H) The effect of GALNT7 overexpression on TAZ protein degradation was assessed in NOZ cells. Cells were transfected with a vector control or a construct overexpressing GALNT7. Following treatment with CHX (50 µM), TAZ protein levels were measured at the indicated time points. The relative TAZ protein levels are expressed as log2 fold change normalized to β-Actin and the initial time point.


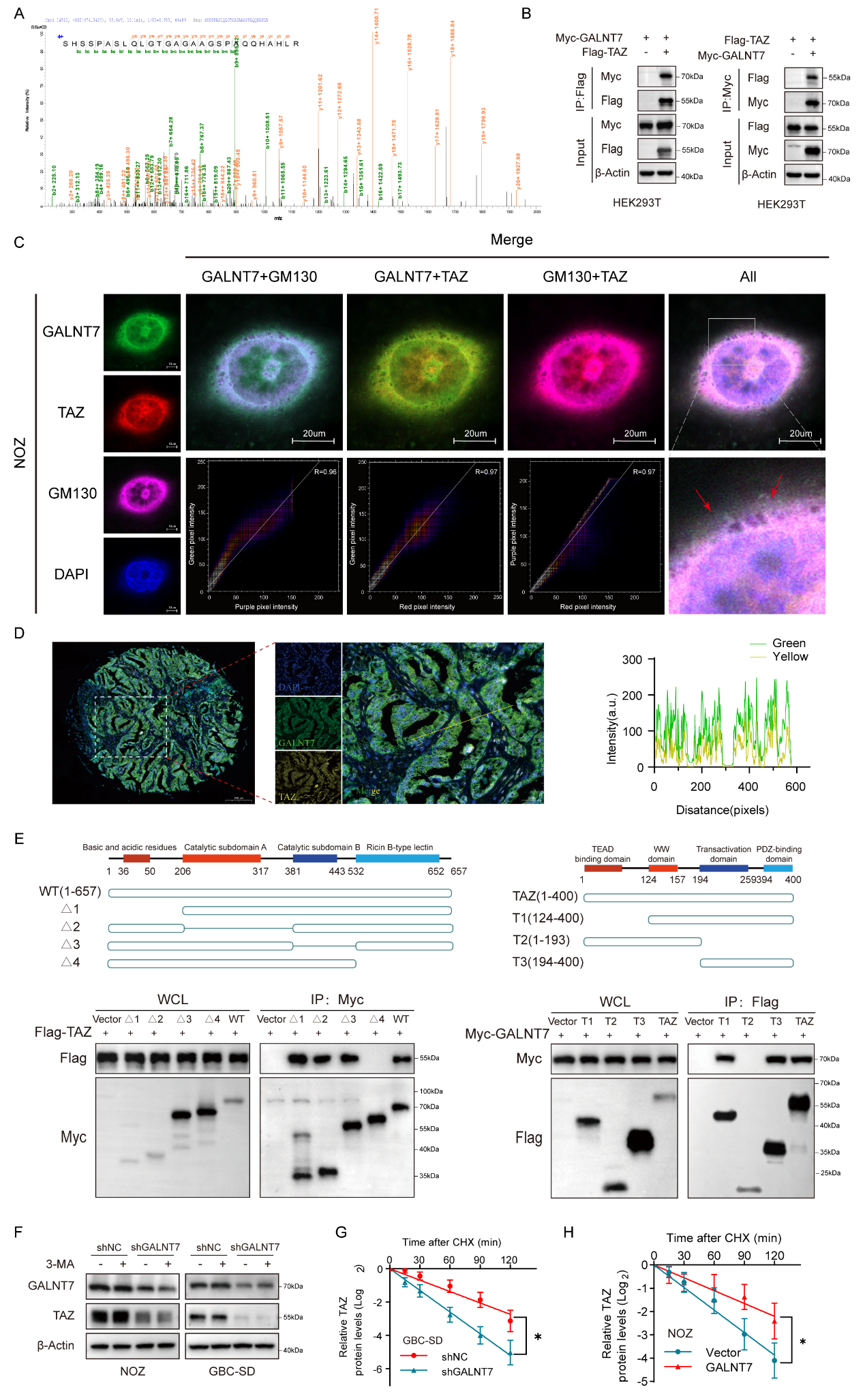


**Figure S3.** **GALNT7 stabilizes TAZ and promotes GBC progression in a glycosyltransferase activity-dependent manner.** (A-B) VVA lectin blotting of whole-cell lysates from NOZ and GBC-SD cells. (A) Global O-GalNAcylation levels were substantially reduced upon GALNT7 knockdown (shGALNT7) compared to shNC controls. (B) Overexpression of GALNT7-WT markedly elevated global O-GalNAc levels, whereas GALNT7-Mut showed no significant effect. (C) Western blot analysis showing that GALNT7-WT significantly increased TAZ protein levels in both NOZ and GBC-SD cells, whereas GALNT7-Mut failed to do so. (D-G) CHX chase assays in NOZ (D, E) and GBC-SD (F, G) cells. GALNT7-WT significantly prolonged TAZ protein half-life, while GALNT7-Mut was unable to prevent TAZ degradation. Quantification of TAZ levels normalized to t = 0 and β-actin is shown in (E) and (G). (H) Ubiquitination assays in HEK293T cells co-transfected with HA-Ub, Flag-TAZ, and Myc-GALNT7-WT or Myc-GALNT7-Mut (MG132, 10 μM, 6 h). GALNT7-WT reduced TAZ ubiquitination compared to GALNT7-Mut. (I, K-L) Functional assays showing GALNT7-Mut lost the ability to promote GBC cell proliferation. Representative images and quantification of colony formation assays (I, L) (n=3) and CCK-8 assays (K) (n=5) in NOZ and GBC-SD cells. **(J, M-N)** Transwell migration and invasion assays showing that GALNT7-WT significantly enhanced cell migration and invasion, whereas GALNT7-Mut failed to do so. Representative images (J) and quantification (M-N) are shown. (O-P) Wound healing assays demonstrating that GALNT7-WT promoted cell migration while GALNT7-Mut did not. Representative images at 0 h and 48 h (O) and quantification (P) are presented. Data are represented as means ± SD. *: p < 0.05, **: p < 0.01, ***: p < 0.001; two‐tailed unpaired Student's t‐test (K-M, P); two‐way ANOVA with Tukey's test (E, G).


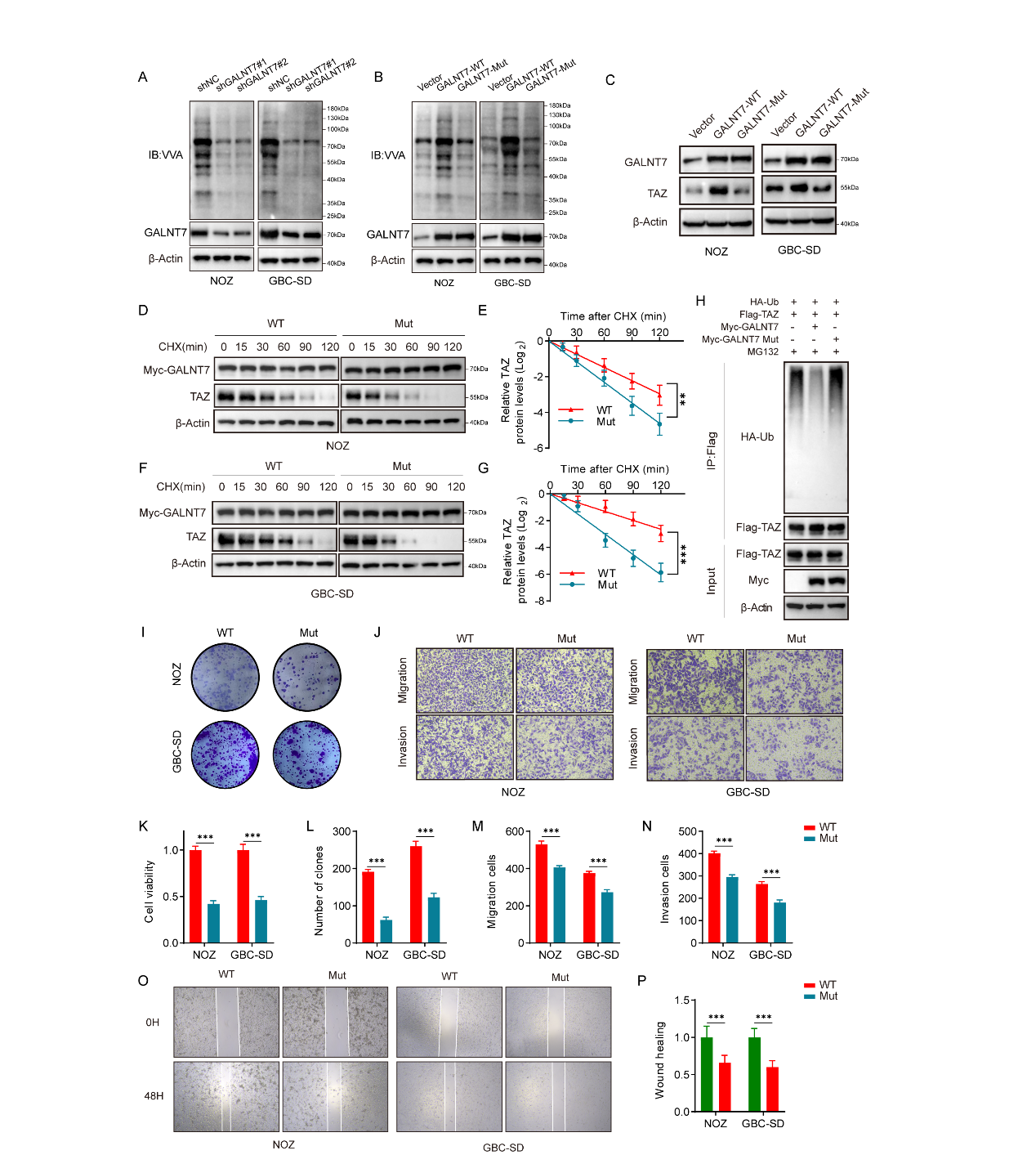


**Figure S4. TAZ S307 O-GalNAcylation promotes the proliferation, invasion, and migration of GBC.** (A) TAZ O-GalNAcylation does not undergo further elongation in GBC-SD cell line. TAZ was immunoprecipitated from GBC-SD cells treated with MG132 and analyzed by lectin blotting. SNA (sialic acid-specific) and PNA (T antigen-specific) showed no binding to TAZ, indicating the absence of glycan elongation. (B)The mass spectrometry analysis revealed the specific site of O-GalNAcylation on the TAZ protein, as identified through spectrographic data. (C)TAZ S307 site aa in different species. (D) Flag-TAZ (WT or S307A) was immunoprecipitated from HEK293T cells and analyzed by immunoblotting with O-GlcNAc-specific antibody (RL2). No O-GlcNAcylation was detected on TAZ. (E) O-glycosidase treatment did not eliminate the immunoreactive band, consistent with the enzyme's inability to cleave unsubstituted Tn antigen (GalNAcα1-O-Ser/Thr). (F) The antibody recognizes Flag-TAZ-WT but not the glycosylation-deficient S307A mutant in reconstituted TAZ-knockout cells, confirming strict specificity for O-GalNAcylated Ser307. (G) TAZ-knockout NOZ and GBC-SD cells reconstituted with Flag-TAZ (WT or S307A) were transfected with or without Myc-GALNT7. Western blot analysis shows comparable levels of p-TAZ Ser89 across all conditions, indicating that O-GalNAcylation does not interfere with LATS1/2-mediated phosphorylation. (H-I) Western blot analysis of TAZ expression post TAZ knockout in two GBC cell lines (H) and expression levels of TAZ protein in NOZ and GBC-SD cell lines transfected with either an empty vector control or a vector expressing wild-type TAZ (WT) or a serine-to-alanine mutant of TAZ (S307A) (I). (J) Colony formation assays to examine the proliferation of two TAZ KO gallbladder cancer cell lines stably expressing Vector, TAZ WT, or TAZ S307A.(K) Cell migration and invasion assays were performed to determine the effects of TAZ and its S307A mutant on the migratory and invasive abilities of two TAZ KO gallbladder cancer cell lines. (L) Wound healing assays were used to evaluate the migratory behavior of two TAZ KO gallbladder cancer cell lines stably expressing Vector, TAZ WT, or TAZ S307A.


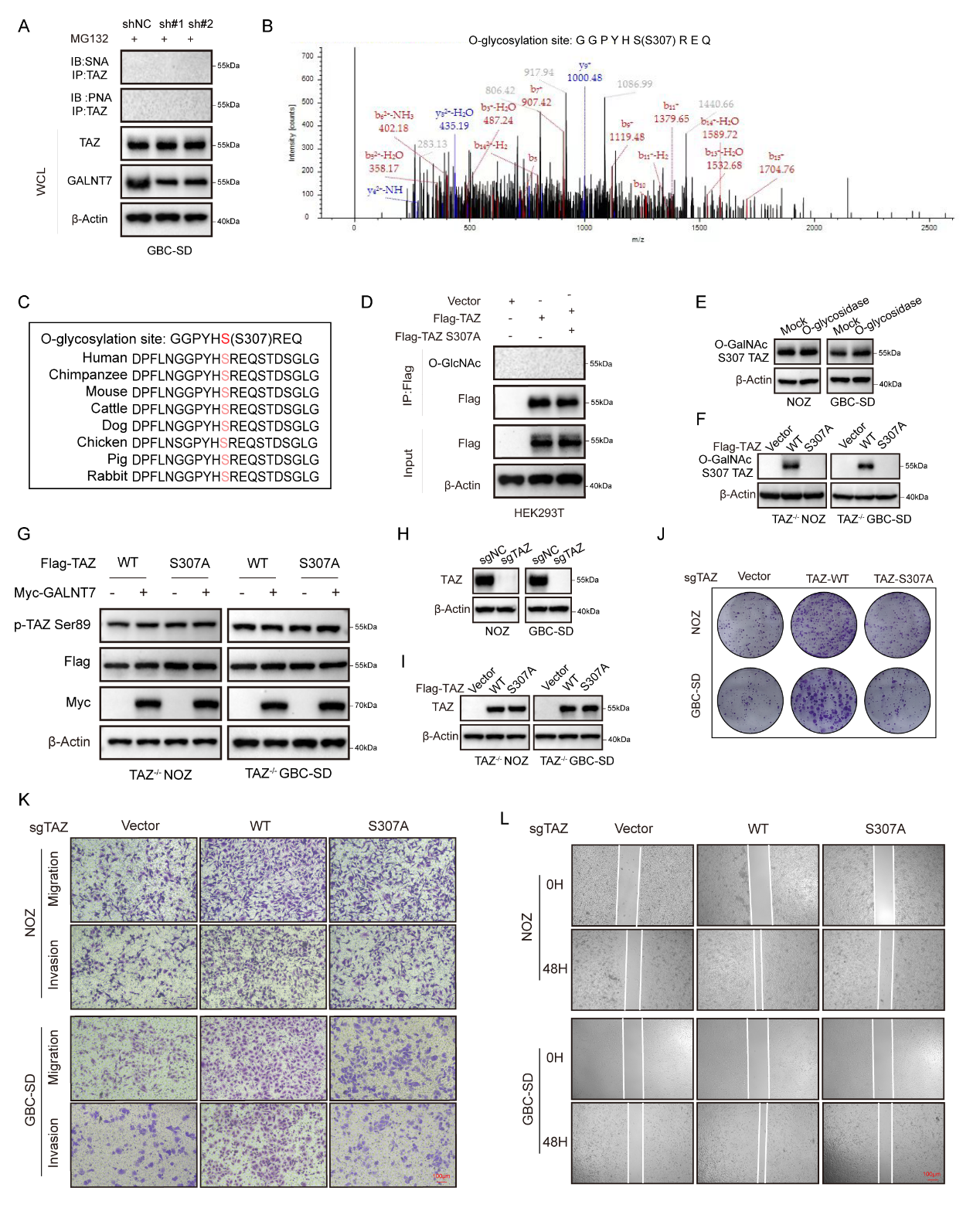


**Figure S5. O-GalNAcylation at Ser307 is required for TAZ-USP7 interaction.** （A）Co-immunoprecipitation from HEK293T cells expressing Myc-USP7, Flag-TAZ (WT or S307A), and HA-GALNT7. The S307A mutation significantly reduces USP7 binding, indicating that Ser307 O-GalNAcylation facilitates the recruitment of USP7 to TAZ.


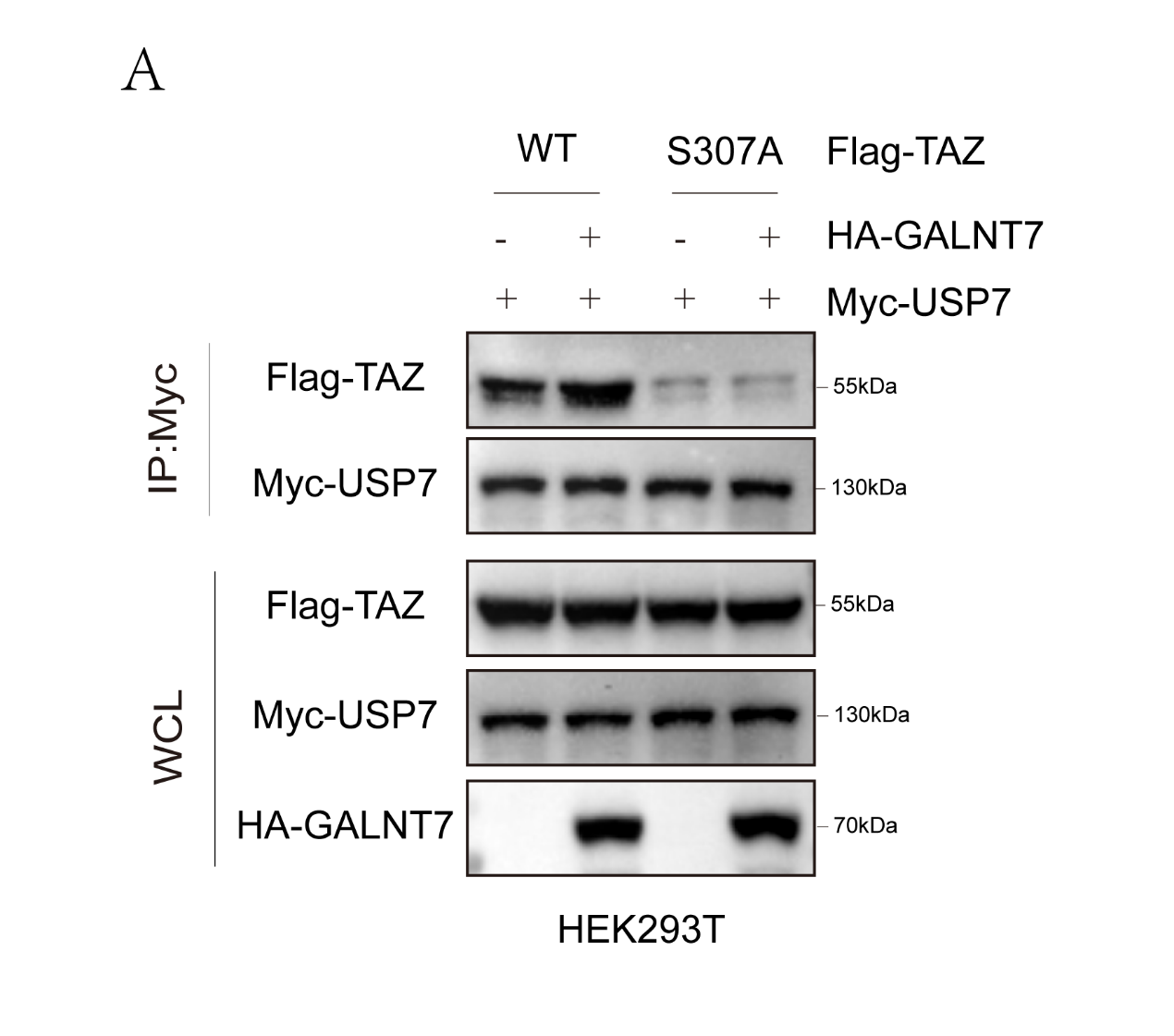
**Figure S6. TAZ S307 O-GalNAcylation is Required for GALNT7-Mediated progression of gallbladder cancer.** (A) Western blot shows Flag-TAZ and Myc-GALNT7 expression in GBC cells. GBC TAZ KO cells were transfected with vectors expressing TAZ WT or TAZ S307A, with or without Myc-GALNT7. (B) Colony formation assays to examine the proliferation of NOZ and GBC-SD TAZ KO cells stably expressing TAZ WT or TAZ S307A and transfected with Vector or Myc-GALNT7. (C) Cell migration and invasion assays were performed to determine migratory and invasive abilities of NOZ and GBC-SD TAZ KO cells stably expressing TAZ WT or TAZ S307A and transfected with Vector or Myc-GALNT7. (D) Wound healing assays were used to evaluate the migratory behavior of NOZ and GBC-SD TAZ KO cells stably expressing TAZ WT or TAZ S307A and transfected with Vector or Myc-GALNT7. (E) Multiple immunohistochemistry (mIHC) analysis was performed on a gallbladder cancer (GBC) tissue microarray comprising 149 patient samples using antibodies against GALNT7, TAZ, and TAZ S307 O-GalNAcylation.


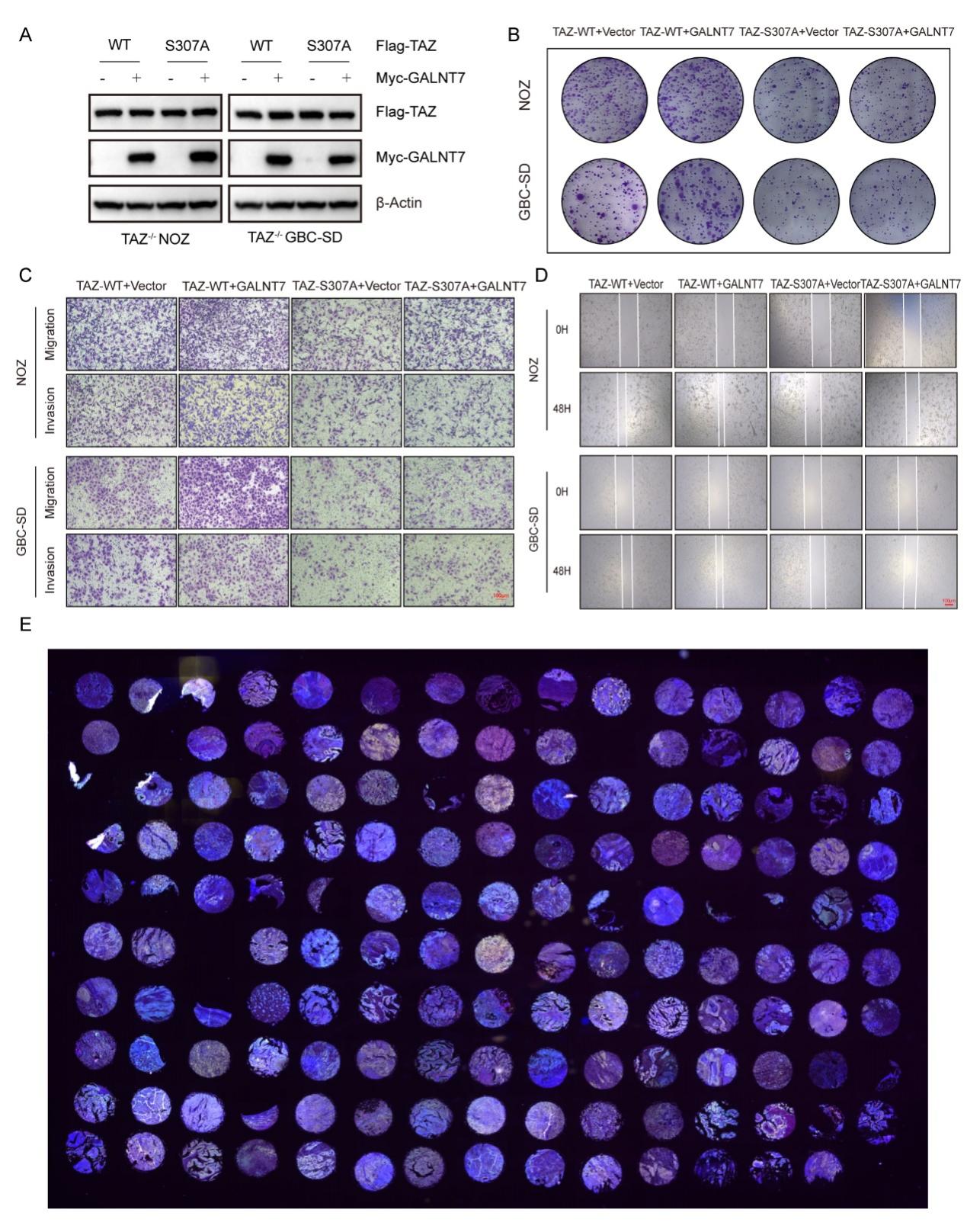


**Figure S7. Identification of Olaparib as a GALNT7-mediated O-GalNAcylation inhibitor that exhibits anti-GBC efficacy. (A)** Colony formation assays to examine the effects of Olaparib (10µM) on cell growth abilities of GBC cells. (B) Cell migration and invasion assays were performed to determine the effects of Olaparib on the migratory and invasive abilities of two gallbladder cancer cell lines. (C) Wound healing assays were used to determine the effects of Olaparib on the migratory behavior of two gallbladder cancer cell lines. (D) Olaparib specifically inhibits TAZ S307 O-GalNAcylation. Western blot analysis of NOZ and GBC-SD cells treated with DMSO, Olaparib, or Niraparib (with MG132). Olaparib markedly reduces TAZ S307 O-GalNAcylation, whereas Niraparib shows no effect. (E) CCK-8 assays were performed to compare the effects of Olaparib (10µM) and Niraparib (10µM) on the proliferation of GALNT7-knockdown and control (shNC) GBC cells. (n = 5 per group). (F) Colony formation assays comparing shNC+Niraparib, shNC+Olaparib, and shGALNT7+Olaparib. Niraparib exhibits minimal effects; GALNT7 knockdown abolishes Olaparib sensitivity. (n = 3). (G-L) Migration, invasion (G-J) and wound healing (K-L) assays showing Niraparib is ineffective, while shGALNT7 renders cells resistant to Olaparib. (n = 3). (M) Dose-response curves of Olaparib in TAZ-knockout cells reconstituted with WT or S307A mutant. Cells expressing S307A exhibit significantly reduced sensitivity, demonstrating that Ser307 O-GalNAcylation is essential for Olaparib efficacy. (N-O) In vivo toxicity assessment in subcutaneous xenograft models showing mouse body weight curves (N) and H&E staining of major organs (heart, liver, kidney). (n = 5 per group). (O) revealing no significant toxicity at 50 mg/kg/day Olaparib treatment in vivo. Data are represented as means ± SD. *: p < 0.05, **: p < 0.01, ***: p < 0.001; one‐way ANOVA with Tukey's test (E, F, I, J, L P); two‐way ANOVA with Tukey's test (M, N).


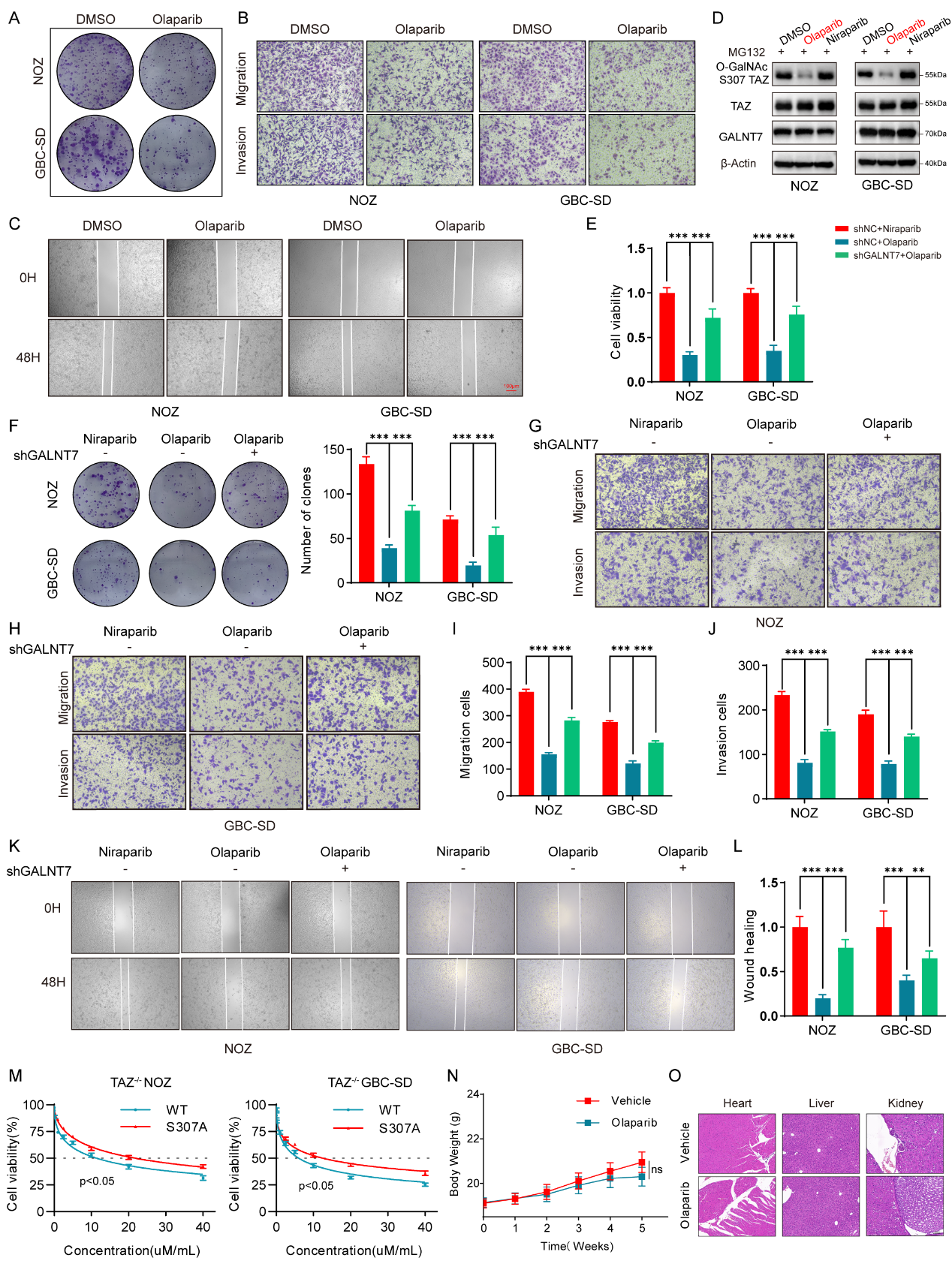
**Table S1. 4D label-free quantitative proteomic profiling of five paired GBC and adjacent non-tumor tissues.**

**Table S2. IP-MS for identifying GALNT7-interacting proteins.**

**Table S3. IP-MS for identifying differential protein between TAZ-WT and TAZ-S307A.**

**Table S4. A virtual-screening campaign identified candidate therapeutics that target GALNT7.**

**Table S5. Correlation between GALNT7 expression and clinicopathologic characteristics of gallbladder cancer patients**

| **characteristics** | **GALNT7 expression** | | **pvalue** |
| --- | --- | --- | --- |
|  | **Low** | **High** |  |
| n | 42 | 58 |  |
| Age（<60 years), n (%) |  |  | 0.0280394330030433 |
| <60 | 26 (26%) | 23 (23%) |  |
| >=60 | 16 (16%) | 35 (35%) |  |
| Gender, n (%) |  |  | 0.356473384880761 |
| Male | 16 (16%) | 17 (17%) |  |
| Female | 26 (26%) | 41 (41%) |  |
| T stage, n (%) |  |  | 1.04067915652362e-05 |
| T1-2 | 21 (21%) | 6 (6%) |  |
| T3-4 | 21 (21%) | 52 (52%) |  |
| N stage, n (%) |  |  | 0.545782813437371 |
| N1-2 | 17 (17%) | 27 (27%) |  |
| N0 | 25 (25%) | 31 (31%) |  |
| AJCC stage, n (%) |  |  | 0.00456048162180002 |
| Ⅲ-Ⅳ | 28 (28%) | 52 (52%) |  |
| Ⅰ-Ⅱ | 14 (14%) | 6 (6%) |  |
| Histological differentiation, n (%) |  |  | 0.00157758298236554 |
| G1 | 10 (10%) | 1 (1%) |  |
| G2-3 | 32 (32%) | 57 (57%) |  |
| Liver metastasis, n (%) |  |  | 1.99144250853072e-08 |
| No | 34 (34%) | 14 (14%) |  |
| Yes | 8 (8%) | 44 (44%) |  |
| CA19-9, median (IQR) | 16.215 (8.54, 62.545) | 123.81 (23.777, 366.73) | 0.000104657909322632 |
| CEA, median (IQR) | 2.64 (1.54, 3.48) | 2.845 (1.6825, 4.71) | 0.343977724471946 |

**Table S6. Univariate and multivariate analysis of various prognostic parameters in patients with gallbladder cancer cox-regression analysis**

| **Characteristics** | **Total(N)** | **Univariate analysis** | | **Multivariate analysis** | |
| --- | --- | --- | --- | --- | --- |
|  |  | **HR (95% CI)** | **P value** | **HR (95% CI)** | **P value** |
| Age（<60 years) | 100 |  |  |  |  |
| <60 | 49 | Reference |  |  |  |
| >=60 | 51 | 1.465 (0.916 - 2.344) | 0.111 |  |  |
| Gender | 100 |  |  |  |  |
| Male | 33 | Reference |  |  |  |
| Female | 67 | 1.625 (0.973 - 2.715) | 0.063 |  |  |
| T stage | 100 |  |  |  |  |
| T1-2 | 27 | Reference |  | Reference |  |
| T3-4 | 73 | 9.011 (4.025 - 20.173) | < 0.001 | 0.710 (0.172 - 2.930) | 0.636 |
| N stage | 100 |  |  |  |  |
| N0 | 56 | Reference |  | Reference |  |
| N1-2 | 44 | 2.366 (1.457 - 3.844) | < 0.001 | 1.128 (0.654 - 1.946) | 0.664 |
| AJCC stage | 100 |  |  |  |  |
| Ⅰ-Ⅱ | 20 | Reference |  | Reference |  |
| Ⅲ-Ⅳ | 80 | 13.818 (4.872 - 39.190) | < 0.001 | 13.556 (2.372 - 77.475) | 0.003 |
| Histological differentiation | 100 |  |  |  |  |
| G2-3 | 89 | Reference |  | Reference |  |
| G1 | 11 | 0.205 (0.072 - 0.579) | 0.003 | 0.613 (0.159 - 2.356) | 0.476 |
| Liver metastasis | 100 |  |  |  |  |
| No | 48 | Reference |  | Reference |  |
| Yes | 52 | 4.681 (2.758 - 7.944) | < 0.001 | 1.346 (0.741 - 2.446) | 0.330 |
| CA19-9 | 100 | 1.000 (1.000 - 1.001) | < 0.001 | 1.000 (1.000 - 1.001) | 0.002 |
| CEA | 100 | 1.003 (1.002 - 1.005) | < 0.001 | 1.003 (1.001 - 1.005) | 0.004 |
| GALNT7 expression | 100 |  |  |  |  |
| Low | 42 | Reference |  | Reference |  |
| High | 58 | 4.057 (2.408 - 6.836) | < 0.001 | 3.540 (1.903 - 6.584) | < 0.001 |

**Table S7. Targeting sequences for shRNAs**

| **shRNAs** | **Sequences** |
| --- | --- |
| sh*GALNT7*#1 | 5'- CGCCCATTTATGTTGGGTCTT -3' |
| sh *GALNT7*#2 | 5'- GCAAATCAACTCATGCAGTAT -3' |
| sh*USP7* | 5'- CCTGGATTTGTGGTTACGTTA -3' |

**Table S8. Information for antibodies used in this study**

| **Protein** | **Assay** | **Catalog number** | **Company** |
| --- | --- | --- | --- |
| β-Actin | WB | 20536-1-AP | Proteintech |
| TAZ | IF，IP | 66500-1-Ig | Proteintech |
| TAZ | WB，mIHC | 23306-1-AP | Proteintech |
| GALNT7 | WB,IP,IF,mIHC | ab254971 | Abcam |
| USP7 | WB | 66514-1-Ig | Proteintech |
| Flag | WB | F7425 | Sigma-Aldrich |
| HA | WB | AE008 | ABclonal |
| Myc | WB | 16286-1-AP | Proteintech |
| TEAD1 | WB,chip | A5218 | ABclonal |
| TEAD2 | WB | A15594 | ABclonal |
| TEAD3 | WB | A7454 | ABclonal |
| TEAD4 | WB | A23774 | ABclonal |
| TAZ S307 O-GalNAcylation | WB,mIHC | Customized antibodies | AtaGenix |

**Table S9. Primers for ChIP-qPCR**

| **Primer set** | | **Primers** | **Sequence (5’-3’)** |
| --- | --- | --- | --- |
| TFBS1 | Forward  Reverse | | 5'- ACAGTCCCTCTTCCAGGTACTCTA -3'  5'-TCAAAATTTCCACAAAATATCTGAAGTGATATCCT -3' |
| TFBS2  TFBS3 | | Forward  Reverse  Forward  Reverse | 5'- AAATTAGCAGTTTAGGATATAGTACACTGATAATGT -3'  5'- GCCTGTGTTACCTAAATTACACAGTAAAGGATATT -3'  5'- GGGCTCGCGTGGAGAG -3'  5'- CCCGTGGCCCCCTC -3' |
